# Nanoparticle Exsolution on Perovskite Oxides: Insights into Mechanism, Characteristics and Novel Strategies

**DOI:** 10.1007/s40820-023-01258-4

**Published:** 2023-11-28

**Authors:** Yo Han Kim, Hyeongwon Jeong, Bo-Ram Won, Hyejin Jeon, Chan-ho Park, Dayoung Park, Yeeun Kim, Somi Lee, Jae-ha Myung

**Affiliations:** Department of Materials Science and Engineering, Incheon National University, Incheon, 22012 Republic of Korea

**Keywords:** Supported nanoparticle, Exsolution, In situ growth, Mechanism, Perovskite oxide, Catalyst

## Abstract

Supported nanoparticles have attracted considerable attention as a promising catalyst for achieving unique properties in numerous applications, including fuel cells, chemical conversion, and batteries. Nanocatalysts demonstrate high activity by expanding the number of active sites, but they also intensify deactivation issues, such as agglomeration and poisoning, simultaneously. Exsolution for bottom-up synthesis of supported nanoparticles has emerged as a breakthrough technique to overcome limitations associated with conventional nanomaterials. Nanoparticles are uniformly exsolved from perovskite oxide supports and socketed into the oxide support by a one-step reduction process. Their uniformity and stability, resulting from the socketed structure, play a crucial role in the development of novel nanocatalysts. Recently, tremendous research efforts have been dedicated to further controlling exsolution particles. To effectively address exsolution at a more precise level, understanding the underlying mechanism is essential. This review presents a comprehensive overview of the exsolution mechanism, with a focus on its driving force, processes, properties, and synergetic strategies, as well as new pathways for optimizing nanocatalysts in diverse applications.

## Highlights

- Fundamental mechanisms in terms of driving force, material design, and exsolution processes are outlined, and novel behaviors of socketing and shape-shifting throughout the interaction with the oxide support are discussed.
- This review examines the key control factors, encompassing external conditions and intrinsic properties that affect the surface exsolution of metallic nanoparticles.
- The extraordinary nature of exsolution particles and their effect on various applications are discussed, along with the latest strategies for improving exsolution behavior.

## Graphical abstract

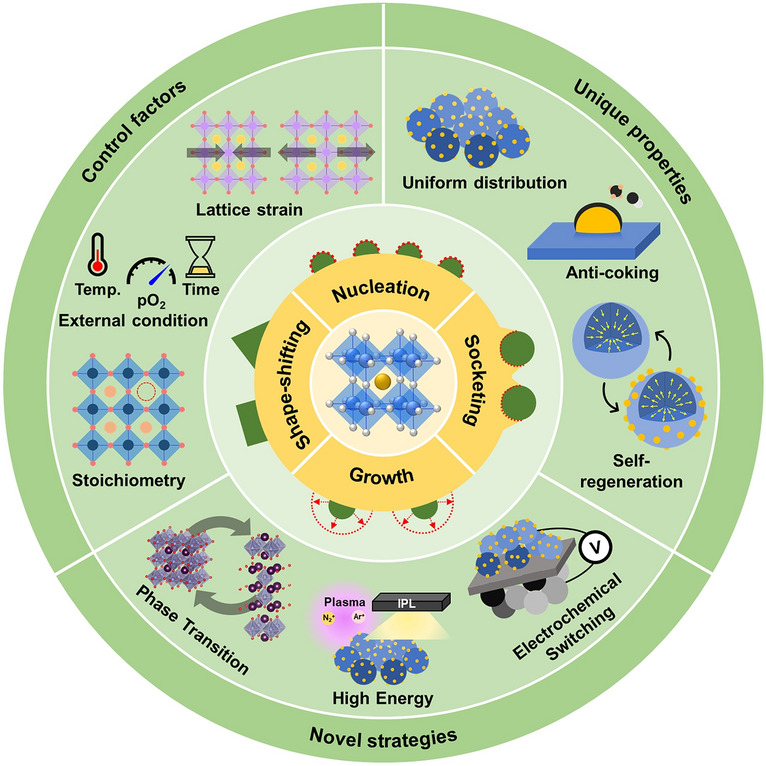

## Introduction

Nanoscale engineering plays a key role in obtaining novel properties for a broad range of applications including electrochemical devices, semiconductors, sensors, and photocatalysis [1–7]. Among nano-engineered materials, supported nanoparticles have received significant attention as a desirable structural concept in heterogeneous catalysis. Active nanoparticles are dispersed across the surface of oxide supports, leading to a drastic improvement in catalytic activity owing to the expanded surface/interface properties (e.g., active surface area and phase boundaries) [8–10]. The preparation of supported nanoparticles has been accomplished by top-down techniques such as wet chemical impregnation and physical/chemical depositions. Unfortunately, these techniques involve complex processes, high costs, and posing significant challenges to their scalability. Uniformity and quality of top-down techniques are affected by the microstructure, scale, and surface toughness of supports, causing limitations such as non-uniform particle distribution and weak adhesion between the particles and the support material. As a result, conventional nanocatalysts suffer from deactivation problems such as agglomeration and poisoning, leading to a shortened operational lifespan.

To overcome these issues, exsolution phenomenon has been explored as an alternative due to its exceptional stability and uniformity [11–14]. Exsolution is a controlled phase separation technique used to uniformly grow nanoparticles on the support. Targeted metals are doped into the host oxide lattice during heat treatment or synthesis and then are exsolved as nanoparticles on the surface from the oxide solid solution under reducing conditions at elevated temperatures. The exsolved nanoparticles are strongly anchored into the oxide support with strong metal-support interaction [15–19]. The socketed nanoparticles on the oxide support show exceptional resistance to agglomeration, carbon coking, and sulfur poisoning, enabling long-term stable operations in diverse energy conversions such as solid oxide cells and reforming catalysis [19–30]. In addition, submerged nanoparticles within the bulk of oxides are often observed after high-temperature reductions [31]. Although these endogenous particles are separated from the reactive gas phases, the self-strained nanostructure of these particles positively impacts the reversible transport and storage of oxygen in redox cycling applications, such as chemical looping [32].

Perovskite oxides are mostly employed as host materials for exsolution because of their stability under redox conditions and high temperatures [33–36] as well as their tunability for doping [37] and non-stoichiometry [38]. Exsolution nanoparticles on perovskite oxide has been controlled by adjusting external conditions and intrinsic factors. The exceptional tunability of exsolvable perovskite oxides plays a pivotal role in broadening their range of applications. Their inherent ionic/electronic conductivity and catalytic activity also make them highly suitable for various energy conversion applications [39–41]. The exsolution process in perovskite oxides is more complex than regular metal separation from alloys due to its dependence on the thermodynamics and kinetics related to phase transition, nucleation, and diffusion/reduction of oxygen ions and cations [42]. Despite significant progress in tailoring exsolved materials by controlling the composition, particle population, size, and shape of nanoparticles, an overall understanding of the exsolution mechanism is still required [43–50].

Recently, numerous studies have extensively explored exsolution phenomena, delving into exsolvable materials, influencing factors, characterizations, and their applications [45, 51]. Most reviews on exsolution phenomena primarily emphasize engineering and optimization for their applications, especially in electrochemical devices [19, 52–56]. Several overviews have focused on mechanisms, including driving force [51], atomic scale [42] and interfaces [57]. However, there still exists a noticeable research gap concerning the fundamental mechanisms underlying the exsolution process and the associated morphologies. In this review, our primary focus is on elucidating the mechanism underlying the nanoparticle exsolution processes taking place in perovskite oxides. We outline various aspects such as the driving force for achieving exsolution, the individual processes, the properties of exsolved nanoparticles, and the synergetic strategies. Firstly, the fundamental mechanism underlying each individual step of the exsolution process, from nucleation to growth, is extensively discussed. Next, we deal with diverse extrinsic and intrinsic factors for controlling the surface exsolution of nanoparticles. Furthermore, the unique properties of exsolved particles caused by the interaction between particle and support are examined. Numerous strategies for enhancing the kinetics and thermodynamic driving force of exsolution are also highlighted. Finally, we offer an outlook on the future prospect of each aspect, including current limitations and overarching goals to provide valuable advancements in exsolution technique.

## Fundamental Mechanism

A comprehensive understanding of the mechanism is crucial for precise control of exsolution; however, due to the complexity of the exsolution phenomenon, our understanding remains incomplete. Exsolution involves a series of processes, including the continuous reduction and migration of oxygen ions and metal cations, followed by nucleation, socketing, and growth. Numerous studies have been conducted to investigate the mechanisms of these processes, which are influenced by external conditions, chemical composition, crystal defects, and nano-microscale defects. We have integrated the individual processes in this chapter and discussed the fundamentals of exsolution, including the driving force and material design for achieving exsolution, while also providing insights into the detailed processes involved in the exsolution phenomenon.

### Driving Force and Material Design

**Fig. 1:**
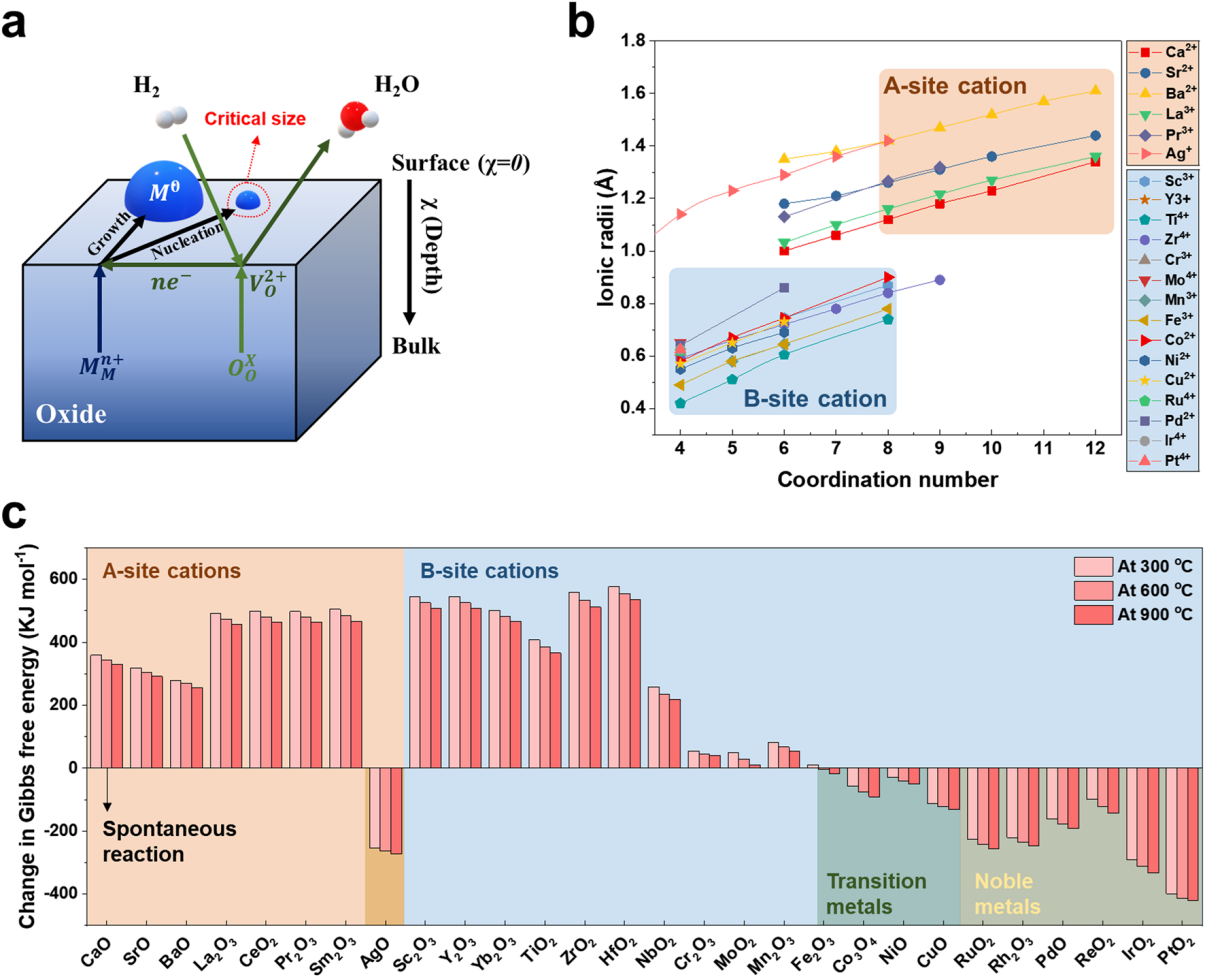
**a** Schematic illustration of exsolution process under H_2_ gas reduction. **b** Ionic radius of metal cation candidates depending on coordination number. **c** Change in Gibbs free energy of oxide reduction reaction ($\frac{1}{x}{\text{M}}_{x} {\text{O}}_{y} + \frac{y}{x}{\text{H}}_{2} \left( g \right) \to {\text{M}} + \frac{y}{x}{\text{H}}_{2} {\text{O}}\left( g \right)$) in H_2_ at 300, 600, and 900 °C in which the energy values are obtained using the HSC chemistry software

In exsolution processes, reduction atmosphere and temperature are key driving forces for triggering phase separation, reduction, and diffusion, followed by nucleation and growth. In terms of thermodynamics, the conditions of sufficiently low pO_2_ and high temperature are essential to achieve $\Delta G_{{{\text{reduction}}}} < 0$ for reducing targeted cations and related oxygen ions. To achieve low pO_2_, numerous studies have controlled gas conditions such as H_2_, CO, CH_4_, ultra-high vacuum, and even H_2_O vapors [34, 58, 59]. In addition, electrical biasing in the electrochemical cells can induce ultra-low pO_2_ conditions and promote the migration of oxygen ions and cations [1]. Figure 1a displays a schematic exsolution process in an H_2_ environment. Upon exposure to reducing conditions, surface reduction in parent oxides takes place in which oxygen vacancies ($V_{{\text{O}}}^{2 + }$) and electrons ($e^{ - }$) are created at the surface (Eq. 1). Continuous surface reduction and diffusion of oxygen ion lead to bulk reduction of oxides. Released electrons reduce exsolvable cations ($M_{M}^{n + }$) in the oxide lattice into metallic states ($M^{0}$), which indicates exsolution (Eq. 2) [60]. For a stoichiometric single perovskite oxide (ABO_3_) as a basic model, emergence of exsolution is started by the reduction in a metal cation, usually a B-site cation, when the concentration of oxygen vacancies ($\delta$) reaches the maximum level ($\delta_{{{\text{lim}}}}$) (Eq. 3) [61]. Hence, the exsolution phenomenon is observed predominantly in metals with higher levels of reducibility. Throughout the exsolution process, it is crucial to maintain the stability of the perovskite structure while preserving the parent phase.

1 $${\text{O}}_{{\text{O}}}^{X} \to V_{{\text{O}}}^{2 + } + 2e^{ - } + \frac{1}{2}{\text{O}}_{2} \left( g \right)$$

2 $$M_{M}^{n + } + ne^{ - } \to M^{0} \left( s \right)$$

3 $${\text{ABO}}_{{3 - \delta_{lim} }} \left( s \right) \to {\text{AB}}_{1 - \beta } {\text{O}}_{3 - \delta } \left( s \right) + \beta {\text{B }}\left( s \right)$$

Thereby, the design of perovskite oxide materials is vital for facilitating the exsolution phenomenon. The positions (e.g., A-site and B-site) of cations are determined by coordination numbers and ionic radius. A-site cations have relatively larger ionic radius and higher coordination numbers than B-site cations (Fig. 1b). Therefore, alkaline earth metals and lanthanides (e.g., Ca, Sr, Ba, La, Pr and Ce) with relatively larger ionic size and lower reducibility ($\Delta G_{{{\text{reduction}}}} > 0$ in H_2_ at 900 °C) have been used for A-site host ion with 12-fold oxygen coordination (Fig. 1c). However, in the case of relatively large lattice sizes, Ce can be utilized as a host for the B-site (e.g., BaCeO_3_). Ag is also well-suited for the A-site in the perovskite structure and exclusively exsolvable because of its high reducibility [62, 63]. Highly reducible noble and transition metals (e.g., Ru, Pd, Ni, Co, etc.) are representative exsolution metals, occupying the B-site. Other B-site ions exhibiting $\Delta G_{{{\text{reduction}}}} > 0$ such as Ti, Mo, and Mn have been employed as the B-site host to achieve redox stability. While Ni is typically preferred for the B-site, its placement in the A-site can also be determined by the synthesis method and lattice structure [15]. Interestingly, Fe is not only stable as a B-site host in low-temperature reduction conditions, but also exsolvable at high temperatures [64].

However, such design approaches using only thermodynamic values for reduction reaction have a gap with the experimental tendency due to the difference in energetics between basic oxides and perovskite oxides. It is generally not possible to directly calculate or measure the energetic aspects of exsolution on perovskite oxide materials. Therefore, various studies have used diverse experimental and calculation techniques to characterize synthesis and exsolution behaviors of perovskite oxides. The synthesis of perovskites and cation doping have been confirmed via X-ray diffraction (XRD) and X-ray absorption spectroscopy (XAS) [65]. The onset temperature of exsolution can be experimentally verified through H_2_-temperature programmed reduction (H_2_-TPR) and microscopies [33, 66]. While the observed onset temperature of Fe exsolution deviated from the theoretical values, a similar trend was observed where the onset temperature was higher compared to other transition metals like Cu [64]. Density functional theory (DFT) calculations have emerged as powerful tools for the design of exsolved materials. Several DFT studies calculated the segregation energy ($E_{{{\text{seg}}}} = E_{{{\text{surface}}}} - E_{{\text{sub - surface}}}$) of exsolvable metals in the perovskite models to verify the favorability of exsolution [67, 68].

### Nucleation

**Fig. 2:**
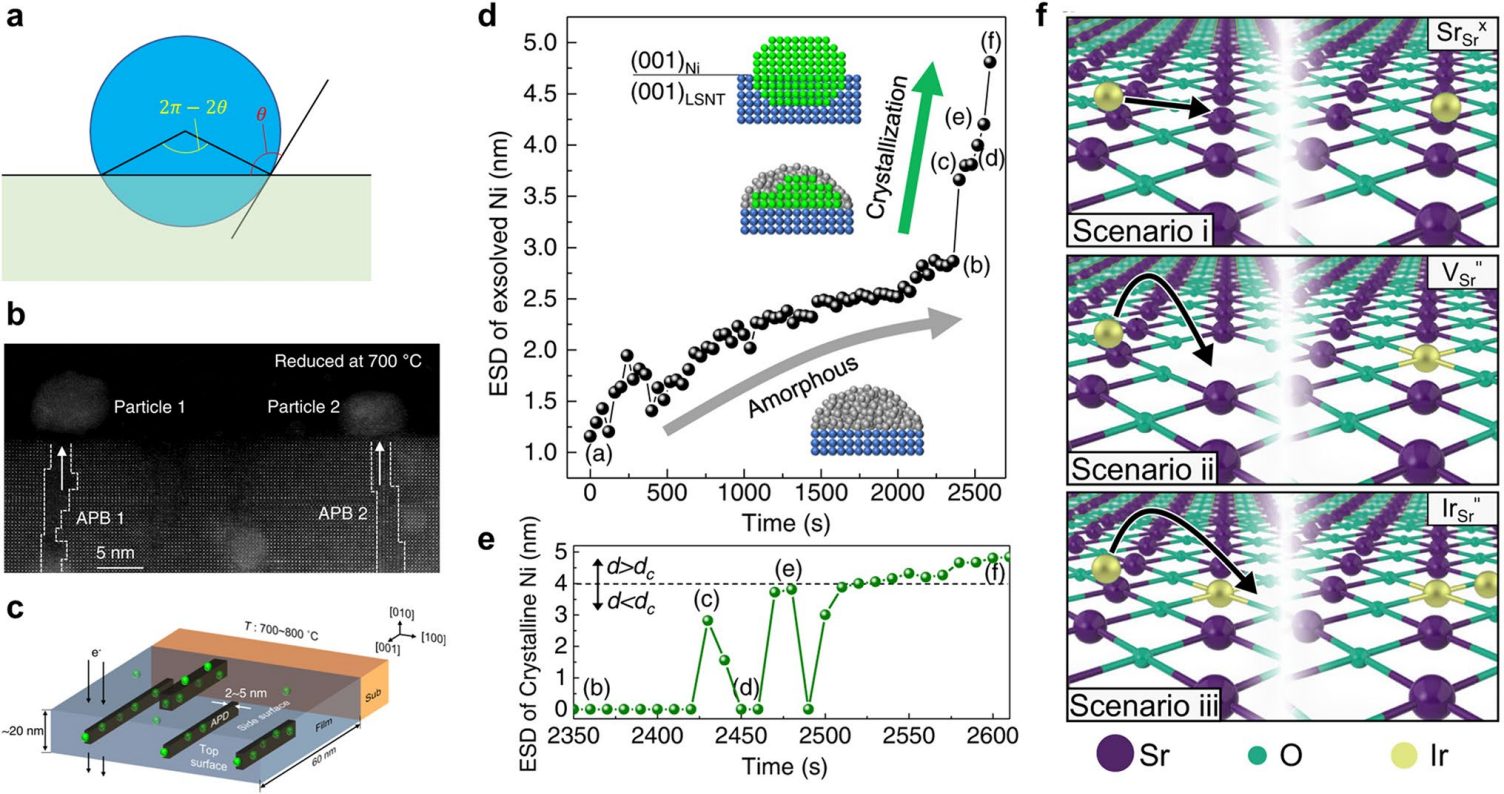
**a** Exsolved nuclei on the oxide surface with a wetting angle. **b** Cross-sectional STEM image of exsolved Ni nanoparticles on the La_0.2_Sr_0.7_Ni_0.1_Ti_0.9_O_3_ _−_ _δ_ after heating treatment inside STEM at 700 °C. **c** Schematic illustration of preferential Ni exsolution near anti-phase boundaries. Time-dependent evolution of size of **d** Ni particle and **e** crystalline Ni. Reprinted permission from Ref. [78]. **f** Scenarios of exsolution process in early stage. Reprinted permission from Ref. [79]

According to classical nucleation theory, the nucleation is explained by Gibbs free energy change of nuclei ($\Delta G_{{{\text{nucleation}}}}$) and the growth is favorable only if the nuclei reach a critical radius ($r^{*}$). For heterogeneous nucleation such as exsolution, $\Delta G_{{{\text{nucleation}}}}$ is related to bulk free energy change ($\Delta G_{{{\text{bulk}}}}$.) per molar volume ($v$) and surface/interface energy terms as follows [69, 70]:

4 $$\Delta G_{{{\text{nucleation}}}} = v\Delta G_{{{\text{bulk}}}} + A_{{{\text{sur}}}} \gamma_{{{\text{sur}}}} + A_{{{\text{int}}}} \gamma_{{{\text{int}}}}^{*}$$

5 $$\Delta G_{{{\text{bulk}}}} = \frac{{ - K_{{\text{B}}} T\ln \left( {1 + \sigma } \right)}}{v}$$

where $A_{{{\text{sur}}}}$ and $A_{{{\text{int}}}}$ are the areas of surface d interface. $\gamma_{{{\text{sur}}}}$ and $\gamma_{{{\text{int}}}}^{*}$ represent the surface and effective interface energies, respectively. $K_{{\text{B}}}$ is the Boltzmann’s constant, T is the temperature, and $\sigma$ is the supersaturation. In general, $\gamma_{{{\text{int}}}}^{*}$ is lower than $\gamma_{{{\text{sur}}}}$, which plays a key role in promoting nucleation kinetics compared to homogenous nucleation. Assuming that a spherical particle is embedded into the support with a certain contact angle ($\theta$) (Fig. 2a), the $\Delta G_{{{\text{nucleation}}}}$ can be expressed as [71]:

6 $$\Delta G_{{{\text{nucleation}}}} = \frac{4}{3}\pi r^{3} \Delta G_{{{\text{bulk}}}} + 2\pi r^{2} \left( {1 - \cos \theta } \right)\gamma_{{{\text{sur}}}} + 2\pi r^{2} \left( {1 + \cos \theta } \right)\gamma_{{{\text{int}}}}^{*}$$

By differentiating $\Delta G_{{{\text{nucleation}}}}$ with respect to $r$ and setting the result is equal to 0, $\frac{{{\text{d}}\Delta G}}{{{\text{d}}r}} = 0$, $r^{*}$ and maximum Gibbs free energy ($\Delta G^{*}$) can be obtained as follows:

7 $$r^{*} = - \frac{{\left( {1 - \cos \theta } \right)\gamma_{{{\text{sur}}}} + \left( {1 + \cos \theta } \right){ }\gamma_{{{\text{int}}}}^{*} }}{{\Delta G_{{{\text{bulk}}}} }} = - \frac{{\gamma^{*} }}{{\Delta G_{{{\text{bulk}}}} }}$$

8 $$\Delta G^{*} = \frac{2}{3}\pi \frac{{\gamma^{*3} }}{{\Delta G_{{{\text{bulk}}}}^{2} }}$$

Using Arrhenius expression, the nucleation rate can be described by Eq. 9:

9 $$\frac{{{\text{d}}N}}{{{\text{d}}t}} = \frac{C}{\tau }{\text{exp}}\left( { - \frac{{\Delta G^{*} }}{{k_{{\text{B}}} T}}} \right) = \frac{C}{\tau } {\text{exp}}\left( { - \frac{{2\pi \gamma^{*3} v^{2} }}{{3k_{{\text{B}}}^{3} T^{3} \left( {\ln \left( {1 + \sigma } \right)} \right)^{2} }}} \right)$$

where *C* is the nucleation site density and $\tau$ is the characteristic timescale [72]. The classical nucleation theory could suggest the importance of Gibbs free energy differences on the nucleation of exsolution nanoparticles. However, there exist some limitations in explaining the distribution of exsolved particles due to its inability to consider the complexity of the exsolution phenomenon. For example, the nucleation sites of exsolution particles are still not fully understood, and particle coalescence can occur during reduction at high temperatures. The surface properties of the oxide, such as crystallinity [73], phase [74], and stoichiometry [38], can undergo changes during exsolution. These pose challenges in predicting and controlling the distribution and population density of the particles. Furthermore, the nucleation of metal relies on a two-step nucleation process involving amorphous nuclei and subsequent crystallization [75]. Thus, classical nucleation theory cannot be directly applied to the nucleation of exsolution particles.

Numerous studies, either experimentally or computationally, have been conducted to elucidate the nucleation mechanism in the exsolution process. It is a recognized fact that grain boundaries and crystal defects can be nucleation site for exsolution, as verified through ex situ experiments [76, 77]. Several efforts have emerged to understand the nucleation process of exsolution through in situ techniques, despite the difficulty of observing nucleation. Neagu et al. [59] verified that the nucleation of Ni nanoparticles on La_0.43_Ca_0.37_Ni_0.06_Ti_0.94_O_3 − δ_ takes place within an extremely short time period of less than 0.2 s and is accompanied by the attainment of a critical size below 1 nm, although instantaneous capture of nucleation was not achieved. A study revealed that during reduction in an A-site deficient perovskite oxide, La_0.2_Sr_0.7_Ni_0.1_Ti_0.9_O_3 − δ_, the nucleation of an exsolved Ni cluster occurs at anti-phase boundaries by providing fast diffusion pathways (Fig. 2b, c) [78]. In addition, they directly observed nucleation and socketing processes in the high vacuum (~ 10^–7^ Torr) using in situ scanning transmission electron microscopy (STEM). The exsolved Ir nanoparticle is formed by “two-step crystallization,” in which the amorphous phase is initially nucleated and then crystallized (Fig. 2d, e). A study using in situ TEM with atomic level resolution and computational techniques investigated the formation process of Ir nanoparticles on SrIr_0.005_Ti_0.995_O_3_ (Fig. 2f) [79]. In the early stages, the Ir^3+^ position is favorable at Sr–Sr bridges on the (001) SrO-termination to interact with O^2−^, suggesting Ir ion movement along (001) SrO-termination. Subsequently, Ir^3+^ is trapped in a Sr vacancy at the surface, and then surface reconstruction takes place by interacting with two Ir ions and forming an Ir–O–Ir pair, providing the initial sites of cluster nucleation. The initial growth accompanied the coalescence of movable clusters and consequently locked them in defective regions, followed by socketing. These results imply that the nucleated particles are strongly socketed at the surface defects with strong metal-support interaction.

### Socketing

**Fig. 3:**
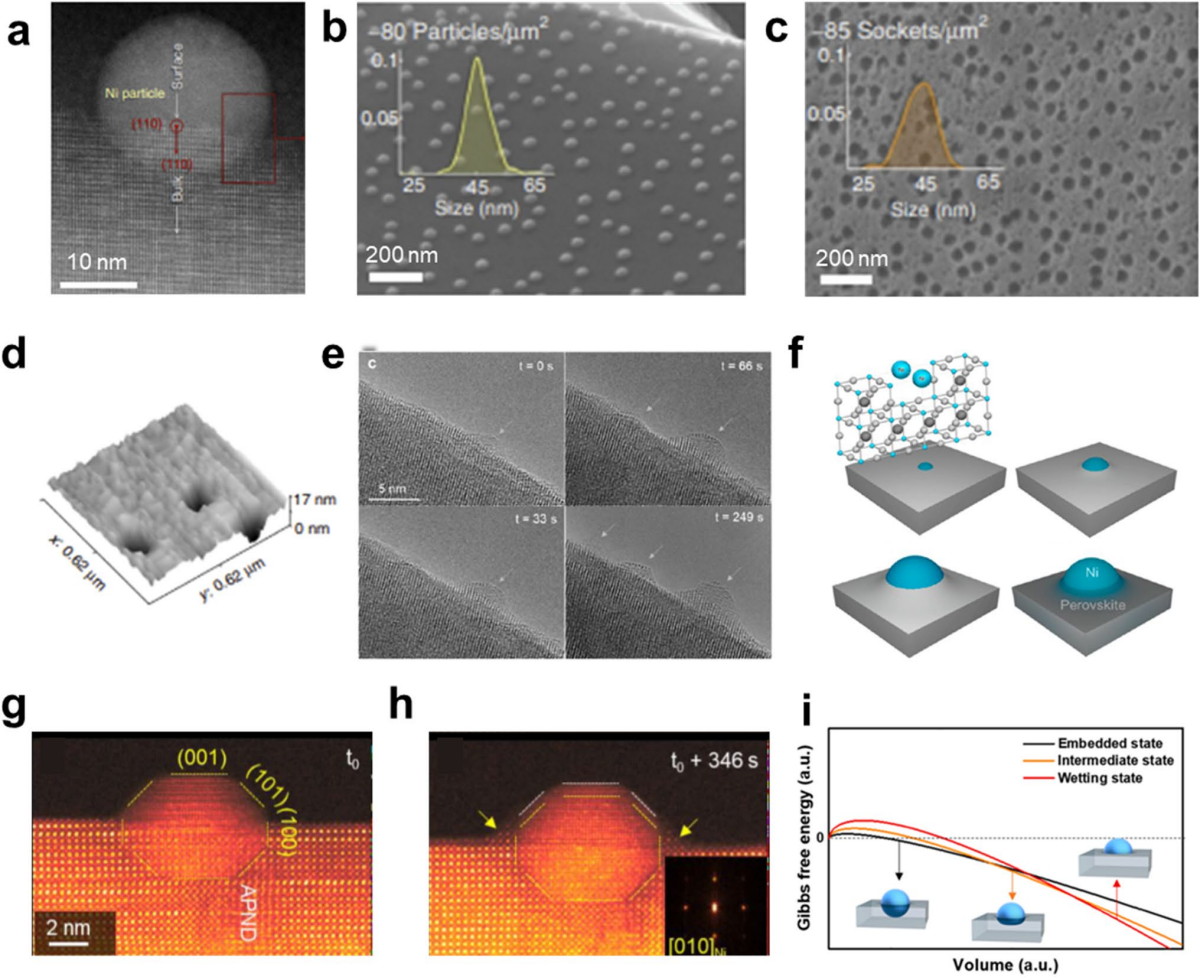
**a** TEM image of an exsolved Ni nanoparticle on the perovskite oxide. Surface morphologies of **b** Ni-exsolved perovskite oxide and **c** a sample etched by HNO_3_. **d** 3D AFM image of a socket after etching. Reprinted permission from Ref. [61]. **e** Environmental TEM analysis of exsolution particles with different reduction time (t) in H_2_. **f** Schematic illustration of nucleation and socketing during particle growth in the exsolution process. Reprinted permission from Ref. [59]. **g**, **h** In situ observation of reactive wetting during growth. Reprinted permission from Ref. [78]. **i** Plot for Gibbs free energies of supported particles with different embedment. Reprinted permission from Ref. [87]

Socketing into the oxide support is one of the unique behaviors in exsolution, affecting catalytic activity and stability. The socketed structure and its embedment have been investigated using microscopies, including scanning electron microscopy (SEM), TEM, and atomic force microscopy (AFM) [80]. As shown in Fig. 3a, atomic-resolved TEM studies revealed that the exsolved Ni nanoparticles are embedded into the surface with an epitaxial relationship between the metal nanoparticles and the host oxides [81]. Neagu et al. [61] demonstrated the ubiquitous formation of socketed structures on the surface by etching the exsolved Ni particles in HNO_3_ (Fig. 3b, c). The sockets on the etched surface have similar population density and size as the Ni particles and this fact was supported by the 3D AFM image, as shown in Fig. 3d.

Recently, various studies have focused on investigating the mechanisms underlying socket formation. Oh et al. [82] analyzed several reduction steps of La_0.4_Sr_0.4_Ni_0.03_Ti_0.97_O_3 − δ_ using AFM. After the first reduction at 600 °C, nanosized pits were observed on the surface of the oxide. Following a second reduction at 700 °C, metallic nanoparticles were formed in the center of each pit and partially embedded. High-temperature reduction at 900 °C resulted in an increase in the height of nanoparticles with a retaining trench structure under the particle. Namely, the nanoparticles were nucleated from underneath the surface and their positions are fixed at surface defects, which can appear during reduction. This process was demonstrated by analyzing particle-support interactions using elastic energy modeling, in which the shape and extent of matrix deformation were simulated. Furthermore, the socket formation mechanism by interaction was examined in a time-resolved TEM study [59]. In the Ni exsolution process in La_0.43_Ca_0.37_Ni_0.06_Ti_0.94_O_3_, the particles undergo growth at the nucleated site without migration. As the particles grow, they apply lateral pressure to the oxide lattice and thus the perovskite oxide surrounds the particle, leading to the formation of a volcano-shaped socket (Fig. 3e, f). Subsequently, the strained socket structure is relaxed by more rising perovskite oxide to minimize surface tension. Han et al. [78] focused on the role of surface/interface tension forces at the triple junction and elastic strain energy caused by lattice mismatch. The nucleated Ni particles on La_0.2_Sr_0.7_Ni_0.1_Ti_0.9_O_3 − δ_ undergo reactive wetting with the oxide support during crystallization, leading to formation of a ridge accompanying the mass transport between particle and support (Fig. 3g, h). This ridge serves to balance the tension forces by minimizing surface tension and is commonly referred to as sockets. However, it is important to note that the study conducted by Han et al. [78] focused on the growth of nanoparticles formed in a specific context, namely at the anti-phase boundaries (APB) of thin films. Whether similar processes occur in bulk materials remains a subject that requires further investigation and confirmation.

Furthermore, the particle size affects not only the socket shape, but also the embedment of the socketed particles. Although it has been commonly observed that the approximate ratio between perovskite lattice uplift and particle diameter is about 1/3, the presence of particles on top of the support is also observed [83–86]. A study reported that the exsolved particles gradually rise as they grow by increasing the reduction temperature (Fig. 3i) [87]. This tendency was explained by the presence of positive bulk free energy related to the dissociation of chemical bonds. However, it has been noted that the correlation between particle size and embedded depth is not entirely uniform and is subject to variations influenced by factors such as the morphology or the orientations of the perovskite surface due to differences in interfacial energy. Despite the challenges associated with achieving substantial depth in cases of high interfacial energy, the precise relationship between socket formation and interfacial energy has yet to be conclusively demonstrated.

### Growth

**Fig. 4:**
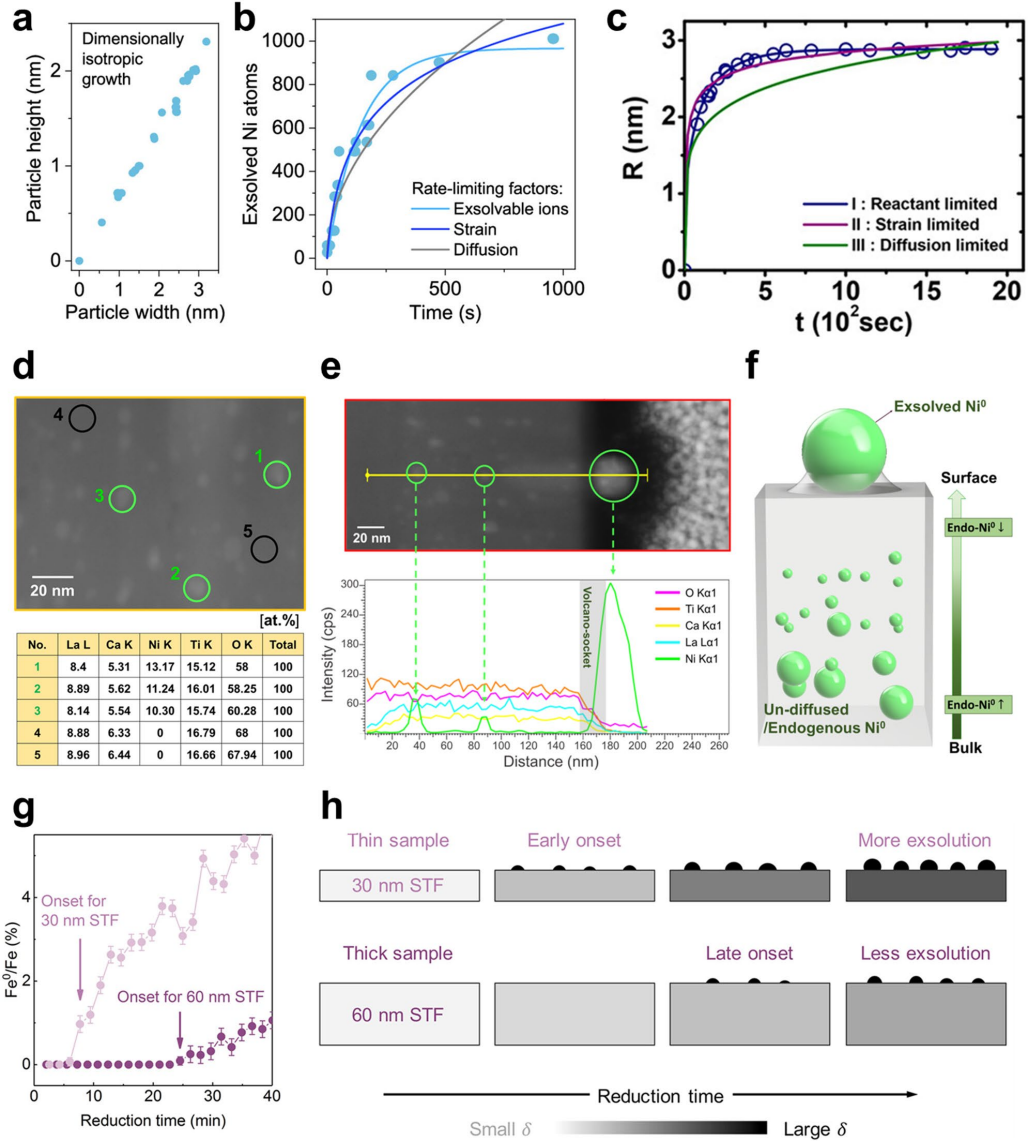
**a** Particle growth with isotropic relation between height and width. **b** The number of exsolved Ni atoms. Reprinted permission from Ref. [59]. **c** Radius of exsolved Co particle as function of time with fitted lines of analytic models. Reprinted permission from Ref. [88]. TEM images of exsolved material with EDS results of **d** points and **e** linear scanning. **f** Corresponding schematics of particle distribution. Reprinted permission from Ref. [90]. **g** Concentration of Fe metal on the surface of thin films with different thickness as a function of the elapsed time. **h** Schematic illustration of exsolution behavior limited by surface reduction. Reprinted permission from Ref. [60]

During the early stages following nucleation, mobile clusters tend to coalesce in order to minimize their surface energy. After socketing, the exsolved nanoparticle is fixed in a region and continuously grows with isotropic behaviors (Fig. 4a) [59]. The growth rate is determined by rate determining steps (e.g., diffusion and reduction). Gao et al. [72] suggested that the growth of exsolved particles is limited by strain, reactant, and diffusion, which were defined as analytic models for size evolution in Eqs. 10, 11, and 12, respectively.

10 $$r_{{\text{s}}} \left( {\text{t}} \right) = r_{{\text{s - lim}}} \left( {\ln \left( {1 + \frac{t}{{\tau_{{\text{s - lim}}} }}} \right)} \right)^{\frac{1}{3}}$$

11 $$r_{r} \left( {\text{t}} \right) = r_{{\text{r - lim}}} \left( {1 - e^{{ - \frac{t}{{\tau_{{\text{r - lim}}} }}}} } \right)^{\frac{1}{3}}$$

12 $$r_{{\text{d}}} \left( {\text{t}} \right) = r_{{\text{d - lim}}} \left( {\frac{t}{{\tau_{{\text{d - lim}}} }}} \right)^{\frac{1}{6}}$$

where $r$ is the radius of a particle as a function of the time ($t$). $r_{{{\text{lim}}}}$ and $\tau_{{{\text{lim}}}}$ are the radius and time constants, respectively. These rate determining steps have been revealed by fitting an analytic model to experimental data. For example, the growth of exsolved Ni particles on a perovskite oxide bulk, La_0.4_Sr_0.4_Ti_0.9_Ni_0.1_O_3 − δ_, under H_2_ environments (~ 1 bar) exhibited good agreement with the strain and reactant limitation models [72]. Furthermore, in situ TEM experiments enabled the quantitative analysis of individual particles as a function of time, providing valuable data on their growth dynamics. The in situ observation of Ni exsolution on a La_0.43_Ca_0.37_Ni_0.06_Ti_0.94_O_3 − δ_ powder under H_2_ (20 mbar) and a high vacuum indicated that the growth is limited by strain/reactant and reactant, respectively, which are caused by locally available exsolvable ions and socketing [59]. Likewise, the size evolution of exsolved Co particles on the polycrystal SrTi_0.75_Co_0.25_O_3 − δ_ thin film was examined within a TEM chamber under high vacuum conditions, which exhibits excellent compatibility with the reactant limitations as shown in Fig. 4c [88]. Time-resolved quantitative analysis of the Fe exsolution on the SrTi_0.65_Fe_0.35_O_3_ thin film was also conducted in the XPS chamber under H_2_ of 0.1 Torr (~ 0.13 mbar) at 400 °C [60]. In this condition, the exsolution process can be clearly divided into two distinct stages. Firstly, the amount of oxygen vacancy in the perovskite oxide reaches the maximum level and subsequently reduction/exsolution of the cation occurs (Fig. 4g). The exsolution of nanoparticle occurs first on the thin film with a lower thickness compared to the relatively thick film, relating to a slower surface reduction rate than the ion diffusion rate (Fig. 4h). This suggests that exsolution under relatively high pO_2_ conditions can be determined by reduction rate.

On the other hand, the nanoparticles have often emerged in the bulk of perovskite oxides at H_2_ conditions, which can also serve as evidence of a slower ion diffusion rate than the reduction rate [89]. Although the individual endogenous particles are smaller than exsolved particles on the surface due to the presence of high strain in the bulk, particle growth on the surface is limited by the diffusion of exsolvable cations and oxygen ions. Kim et al. [90] observed that after reduction in pure H_2_ at 900 °C for 12 h, the endogenous Ni particles in (La,Ca)_1-α_(Ni, Ti)O_3_ thin film were more distributed in the inner bulk rather than near the surface, which indicates cation diffusion (Fig. 4d–f). This suggests that cation diffusion plays a key role in determining the growth kinetics of surface exsolution. In the case of exsolution depending on Ni doping level and reduction temperature/time, the tendency of exsolved Ni atoms on the surface was consistent with the cation diffusion limitation model. The growth limitation in the exsolution processes was diverse in each case. Unavoidably, each experimental methodology and condition, including the type and composition of the sample and reduction conditions, were different. Namely, the rate determining step of particle growth can vary depending on the sample, targeted particle, and reduction condition. Further studies are needed to comprehensively understand the growth limitations based on the reduction conditions and composition requirements. If utilized effectively, this enables quantitative control of particle growth in the exsolution phenomenon.

### Shape-Shifting

**Fig. 5:**
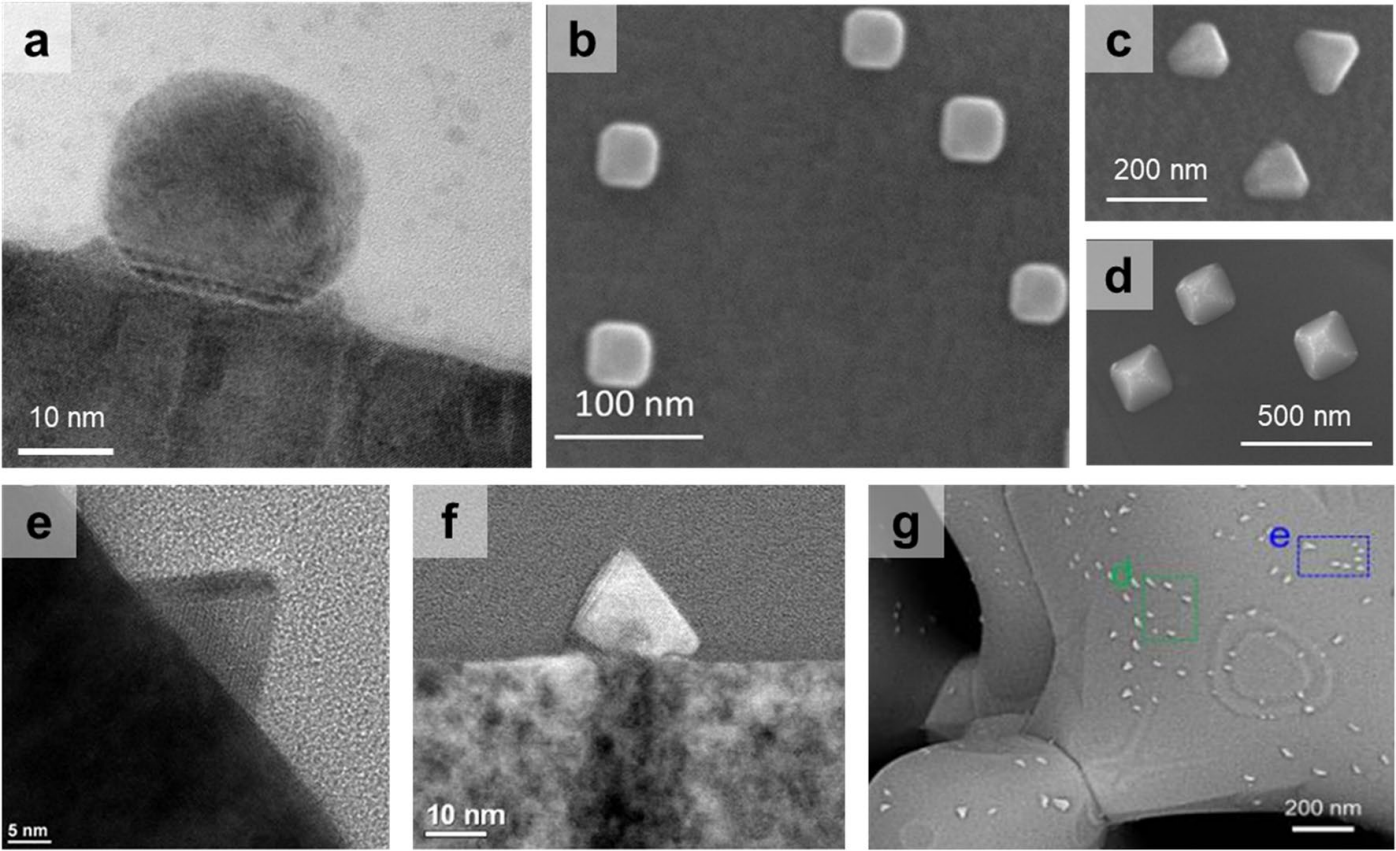
Morphologies of reported metallic exsolution particles with different shapes. **a** Spherical Ni. Reprinted permission from Ref. [90]. **b** Cubic Ni. Reprinted permission from Ref. [59]. **c** Triangular Ni. **d** Pyramidal Ni. Reprinted permission from Ref. [87]. **e**, **f** Cone-shaped CoFe alloy. Reprinted permission from Ref. [42]. **g** Triangular Pt. Reprinted permission from Ref. [65]

Shape control of nanoparticles has emerged as an important technique to achieve precisely tailored catalysts [91–93]. Until now, the shape of exsolution particles has been predominantly described as a quasi-spherical morphology as shown in Fig. 5a. Recently, several studies demonstrated that the exsolved particles can undergo shape-shifting under controlled reduction conditions such as reduction temperature, time and atmosphere. Research found that control of the reducing atmosphere, specifically the gas type and pO_2_, affects the shape of nanoparticles. For La_0.8_Ce_0.1_Ti_0.6_Ni_0.4_O_3_, reduction in a 5%CO atmosphere at 900 °C resulted in a transformation in the shape of Ni nanoparticles to a {100}-faceted cubic shape, unlike reduction in H_2_ (Fig. 5b) [59]. Other shapes of exsolved Ni particles have also been reported as shown in Fig. 5c–g.

**Fig. 6:**
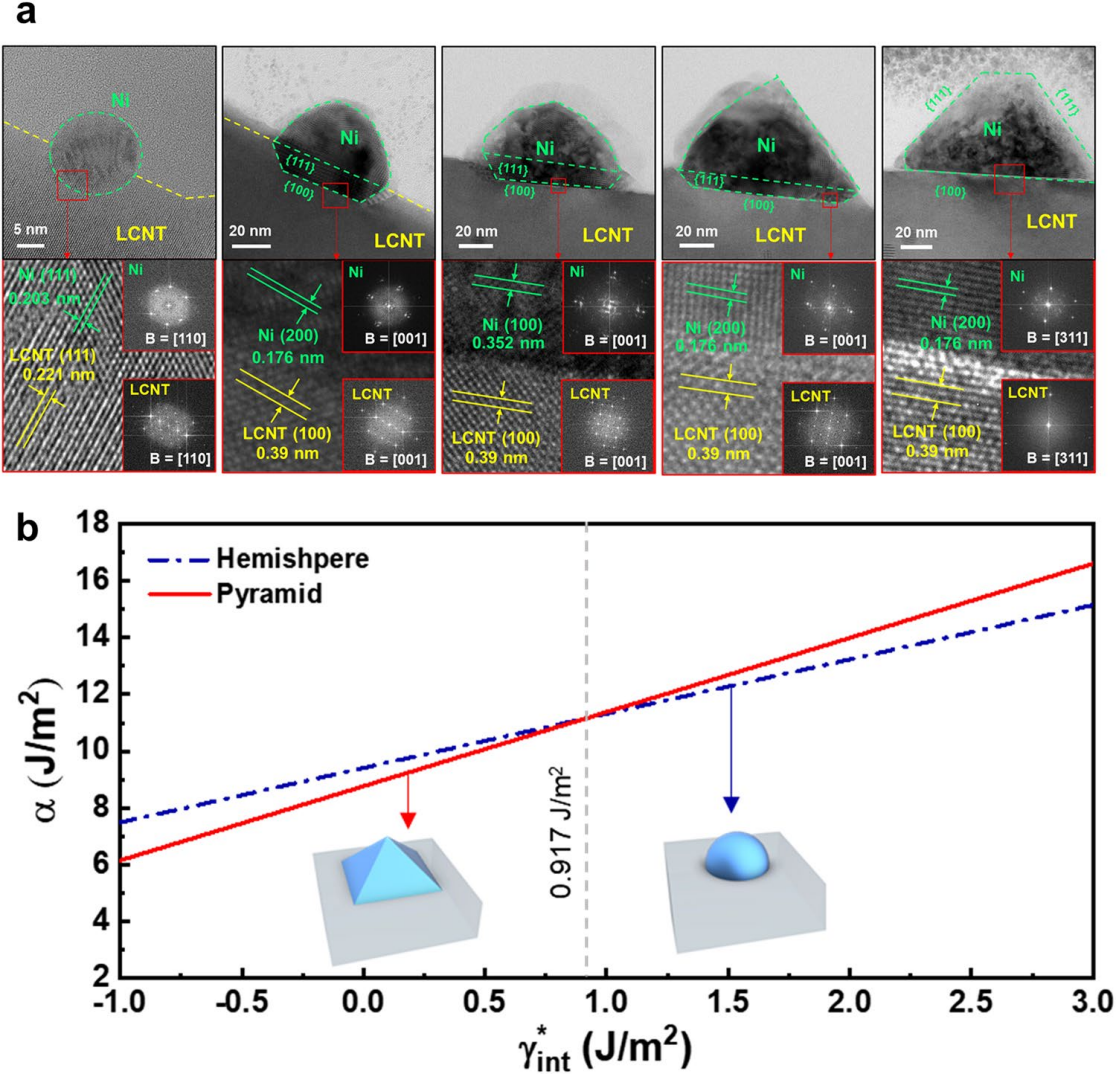
**a** Cross-sectional morphologies of exsolved Ni particles with different size. **b** Plot of shape factor as function of interfacial energy. Reprinted permission from Ref. [87]

Kim et al. [87] investigated the relationship between particle growth and shape-shifting by adjusting reduction conditions using H_2_. As the reduction temperature and time increased, the average size of the Ni particles exsolved on the surface of La_0.7_Ca_0.2_Ni_0.25_Ti_0.75_O_3_ became larger. During particle growth, initial spherical particles were gradually turned into polyhedral shapes (e.g., pyramid and triangular plate) enclosed by the {111} facet (Fig. 6a). This shape-shifting of supported particles was explained by a thermodynamic approach using the shape factor ($\alpha_{{{\text{shape}}}}$) in which the surface/interface energy terms of the particle shape are considered as follows:

13 $$\Delta G_{{{\text{area}}}}^{*} = A_{{{\text{sur}}}} \gamma_{{{\text{sur}}}} + A_{{{\text{int}}}} \gamma_{{{\text{int}}}}^{*} = \alpha_{{{\text{shape}}}} v^{\frac{2}{3}}$$

14 $$\alpha_{{{\text{shape}}}} = \frac{{A_{{{\text{sur}}}} \gamma_{{{\text{sur}}}} + A_{{{\text{int}}}} \gamma_{{{\text{int}}}}^{*} }}{{v^{\frac{2}{3}} }}$$

where $\Delta G_{{{\text{area}}}}^{*}$ is the area free energy. $\alpha_{{{\text{shape}}}}$ is determined by the influence of surface/interface energies and shape. For comparison of various shapes, the lower value of $\alpha_{{{\text{shape}}}}$ indicates a more stable shape. To obtain faceted shapes with larger surface and interface areas compared to spheres, it is crucial to have significantly low interface energy, as shown in Fig. 6b. The interface energy of Ni and a perovskite oxide with epitaxy was calculated to be − 0.02 and − 0.12 J m^−2^, indicating that a faceted shape is energetically favorable.

These findings demonstrate that the interaction between particles and support and the stabilization of surface facets play a significant role in shaping the exsolved particles. These can be affected by various aspects, such as the crystal structure/composition of particles and surface orientation of oxides. To verify tendencies, characteristics of reported exsolution particles with various shapes are summarized in Table 1. As aforementioned, quasi-spherical shapes were readily observed because of their low surface-to-volume ratio, which is favorable for minimizing surface energy. Several studies reported pyramid and triangular plate shapes, which are representative shapes enclosed by the most stable {111}. The formation of these shapes depends on the specific interface facets, such as {100}, {111} facets between particles and support. Although {100} is not the most stable facet for FCC metal, cubic particles with {100}-faceted surface were also observed following reduction, particularly under CO gas conditions. This observation implies that carbon monoxide (CO) gas could have played a stabilizing role similar to that of capping agents for the {100}-facet. In the reported research, it was found that faceted shapes were more commonly observed in larger exsolved particles. To achieve shape-shifting in smaller particle sizes (< 30 nm), it is essential to conduct research focused on stabilizing the surface and interface of exsolved particles. Moreover, research verifying the in situ process of shape-shifting is needed to investigate the mechanism of shape-shifting. Further investigations are warranted to explore and advance the shape-control of exsolution particles.

**Table 1:** Reported exsolution particles with diverse morphologies

Shape	Perovskite oxide		Exsolved metal				Reduction condition	References
	Composition	Surface facet	Composition	Surface facet	Crystal structure	Particle size (nm)		
Quasi-sphere	La_0.7_Ca_0.2_Ni_0.25_Ti_0.75_O_3_	–	Ni	–	FCC	12.5	H_2_, 600 °C, 24 h	[87]
		–	Ni	–	FCC	17.1	H_2_, 700 °C, 24 h,	
		–	Ni	–	FCC	29.2	H_2_, 800 °C, 24 h	
	La_0.9_Sr_0.1_Cr_0.8_Ni_0.2_O_3 − δ_	{201}	Ni	–	FCC	20–50	H_2_, 850 °C, 9 h	[94]
	(La_0.2_Sr_0.8_)_0.95_Ti_0.85_Mn_0.1_Ni_0.05_O_3+δ_	{110}	Ni	–	FCC	60	5%H_2_/Ar, 800 °C, 20 h	[95]
	La_0.2_Sr_0.7_Ti_0.9_Ni_0.1_O_3 − δ_	{100}	Ni	–	FCC	40 ± 17	H_2_, 900 °C, 12 h	[71]
		{110}	Ni	–	FCC	31 ± 9	H_2_, 900 °C, 12 h	
		{111}	Ni	–	FCC	20 ± 7	H_2_, 900 °C, 12 h	
	(La_0.3_Ca_0.7_)_0.95_Fe_0.7_Cr_0.25_Ni_0.05_O_3 − δ_	–	Fe–Ni	–	FCC	36	5%H_2_/N_2_, 750 °C, 5 h	[96]
		–	Fe–Ni	–	FCC	46	5%H_2_/N_2_, 750 °C, 25 h	
Pyramid	La_0.7_Ca_0.2_Ni_0.25_Ti_0.75_O_3_	{100}	Ni	{111}	FCC	82.0	H_2_, 900 °C, 24 h	[87]
		{100}	Ni	{111}	FCC	153.6	H_2_, 1000 °C, 24 h	
Triangular plate	La_0.7_Ca_0.2_Ni_0.25_Ti_0.75_O_3_	{111}	Ni	{111}	FCC	52.1	H_2_, 900 °C, 24 h	[87]
		{111}	Ni	{111}	FCC	155.3	H_2_, 1000 °C, 24 h	
	La_0.65_Sr_0.3_Cr_0.85_Ni_0.15_O_3 − δ_	–	Ni	–	FCC	100	H_2_, 900 °C, 3 h	[97]
	La_0.4_Sr_0.3925_Ba_0.0075_Pt_0.005_Ti_0.995_O_3_	–	Pt	–	FCC	35	5%H_2_/Ar, 700 °C, 12 h	[65]
Cubic	(La_0.3_Ca_0.7_)_0.95_Fe_0.7_Cr_0.25_Ni_0.05_O_3 − δ_	–	Fe–Ni	–	FCC	55	5%H_2_/N_2_, 800 °C, 5 h	[97]
		–	Fe–Ni	–	FCC	104	5%H_2_/N_2_, 800 °C, 10 h	
	Sr_2_Fe_1.3_Co_0.2_Mo_0.5_O_6-δ_	–	Co	–	FCC above 422 °C	134	50%CO/CO_2_, 850 °C, 10 h	[98]
	La_0.8_Ce_0.1_Ti_0.6_Ni_0.4_O_3_	{100}	Ni	{100}	FCC	45.3	5%CO, 900 °C, 10 h	[59]

## Control Factors of Exsolution

Numerous studies on exsolution catalysts are mainly underway to maximize the electrochemical active surface areas in order to improve the catalytic properties for various systems [8]. The fundamental understanding on the main control factors of their surface exsolution properties is believed to play a crucial role in the optimization of exsolution catalysts. Recent studies indicated that the surface morphologies of exsolved nanoparticles, including their size, populations, and shapes, could be determined by external exsolution conditions and the inherent properties of perovskite mother phases. The following sections demonstrate several studies on external and inherent properties affecting surface exsolution.

### External Condition

The external conditions of exsolution, including the reduction temperature, time and pO_2_, determine the surface morphology of exsolved nanoparticles. The exsolution process could be classified into four steps, which involve surface reduction, diffusion, particle nucleation, and growth [72]. The correlation between the nanoparticle nucleation and reduction conditions could be theoretically described by classical isothermal nucleation and growth theory [34, 99]. Equation 9 indicates that the nucleation rate is increased under a high-temperature reduction process; however, practical limitations between theoretical and experimental results are remained due to the complexity of exsolution phenomena. Nevertheless, a gap exists between theoretical predictions and experimental results due to the complexity of exsolution phenomena. To understand exsolution behaviors, several studies have experimentally and analytically investigated the exsolution tendency under different reduction temperature, time, and partial pressure of gases.

**Fig. 7:**
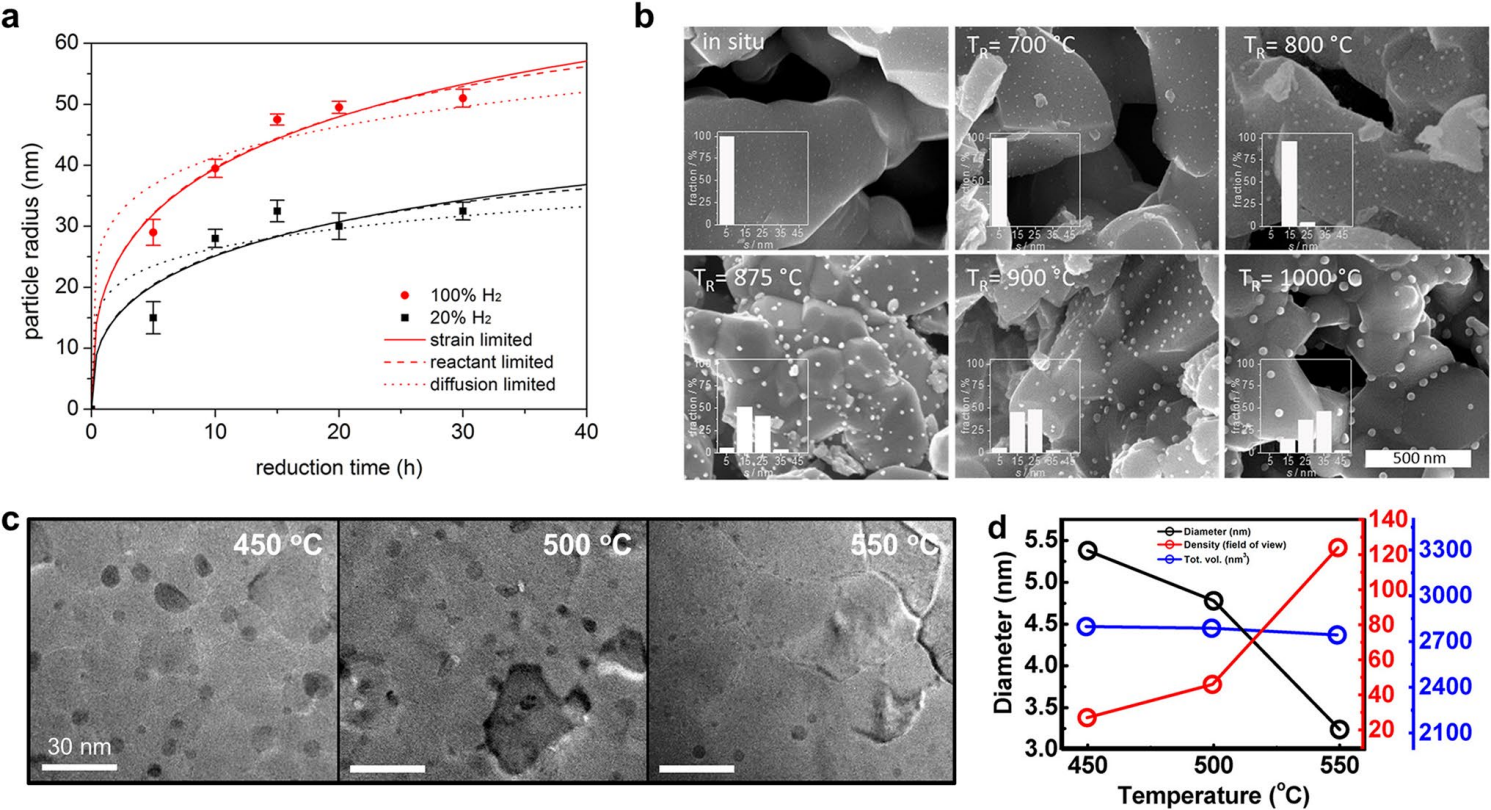
**a** Theoretical and experimental exsolution results on different reduction time and PO_2_ condition. Reprinted permission from Ref. [72]. **b** Exsolution characteristics under different reduction temperatures. Reprinted permission from Ref. [34]. **c** Morphologies of exsolved Co particles in the STF thin film after reduction at 450–550 °C. **d** Corresponding particle tendency regarding size, population density, and the exsolution extent. Reprinted permission from Ref. [103]

Gao et al. [72] introduced H_2_ concentration- and reduction time-dependent exsolution tendencies and their fitting with analytical models. Figure 7a exhibits the theoretical and experimental results of exsolved particle size under different reduction times and H_2_ concentrations. The results demonstrate that the average diameter of exsolution nanoparticles increases as the reduction time prolongs and gradually stabilizes due to growth limitations such as a lack of reactants and diffusion constraints. The limitation factors can be evaluated from saturation tendencies using analytic models (Eqs. 10–12). In this case, the size of exsolved Ni nanoparticles was determined by strain and reactant limitation models. The limited value ($r_{{{\text{lim}}}}$) of particle size can be estimated through this approach [88]. The other result obtained here suggests that under lower pO_2_ conditions resulting from a high H_2_ concentration, there is a tendency for an increase in the average particle size of exsolved nanometal catalysts. The lower pO_2_ condition facilitates the surface reduction in cations, which enables the additional growth of exsolved nanometal catalysts [59, 100–102]. However, the theoretical relationship between particle size and pO_2_ has not been definitively established to date. On the other hand, it was found that the population density of exsolved nanoparticles was affected by the pO_2_ level, with stronger reducing conditions (low pO_2_) exhibiting a higher nucleation rate [1, 103]. Abovementioned exsolution studies with diverse pO_2_ condition usually adopt Ar or N_2_ diluted reducing gas to simply control its reducing atmosphere. The nucleation of exsolution nanoparticle starts within few seconds after starting the reducing process, and the precious control of pO_2_ at the starting point is believed to be crucial in analyzing the effect of reducing pO_2_ on exsolution characteristics. Therefore, the more precious reducing systems, including H_2_O buffered hydrogen reducing system, is required to provide reproducible results on this study.

The size and population of exsolved nanoparticles could be controlled by tailoring the reduction temperature. Neagu et al. observed the size and distribution of exsolved nanoparticles prepared under at different reduction temperatures (Fig. 7b). The temperature during exsolution affects diverse processes including ion diffusion, reduction, nucleation, and coalescence. According to classical theories, diffusion, reduction, and nucleation rates exponentially increase with increasing temperature. Thus, the average size of surface exsolved particles tends to increase as the reduction temperature increases [34, 104]. The surface exsolved particles prepared under lower temperatures showed reduced particle size as well as a higher distribution. However, the obtained results contradict the anticipated relationship between the nucleation rate and high-temperature reducing conditions, as depicted in Eq. 9. Similar results were also observed in diverse perovskite systems [4, 21, 105]. This can be attributed to the increased coalescence of mobile clusters at high temperatures in the early stages prior to socketing, as outlined in the nucleation section. The exsolution tendency between population and temperature is opposite in low-temperature conditions. Kim et al. [103] have explored the exsolution tendency on the Sr_0.98_Ti_0.95_Co_0.05_O_3 − δ_ (STC) thin film under a range of temperatures (450–550 °C) in the H_2_O buffered H_2_ condition at a pO_2_ of 10^–28^ atm (Fig. 7c, d). In this condition, it was observed that the population density of exsolved particles increased as the reduction temperature was elevated, corresponding with nucleation theory.

### Stoichiometry

**Fig. 8:**
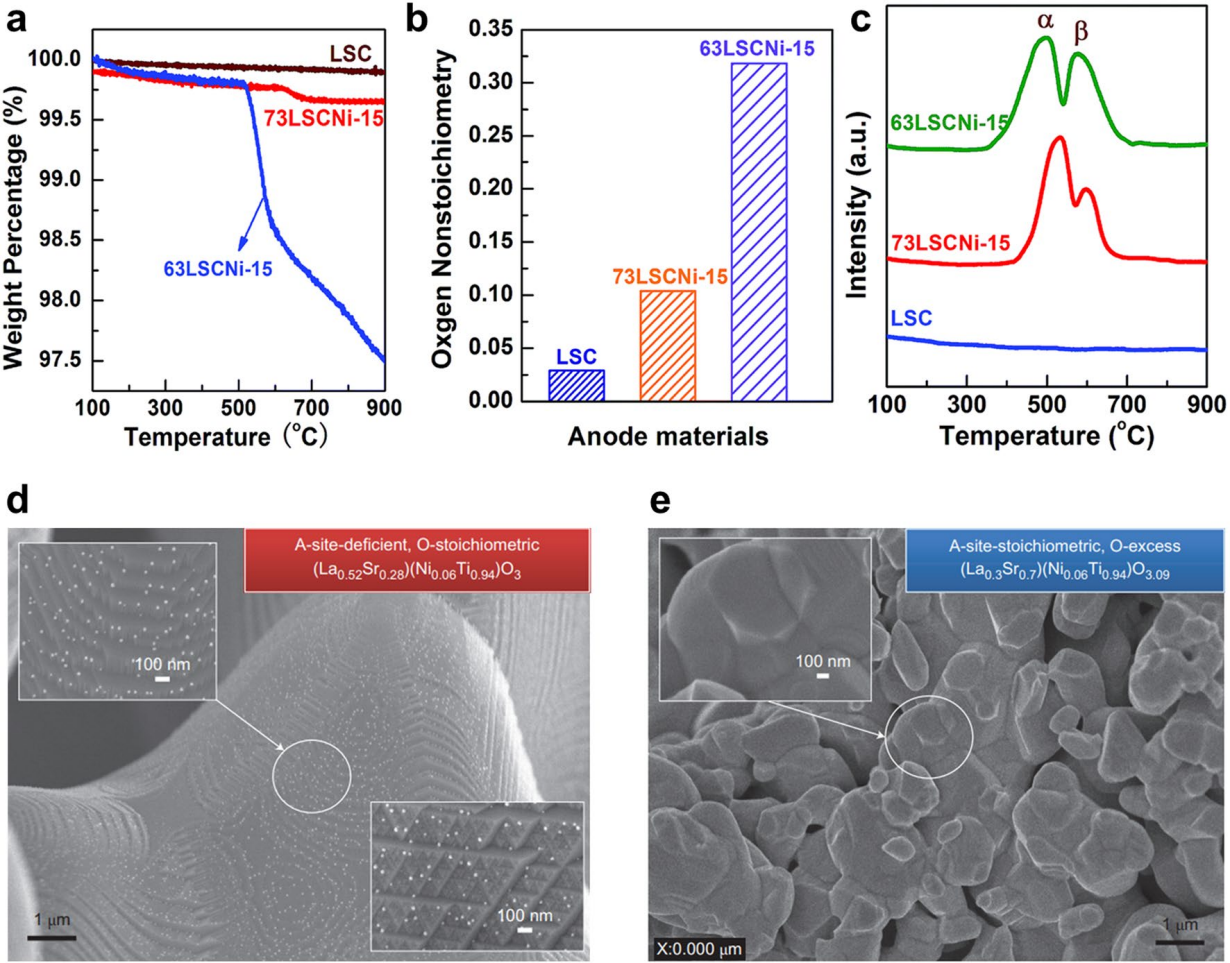
Effect of stoichiometry on exsolution perovskite design. **a** Thermogravimetric analysis (TGA), **b** the extent of oxygen vacancy formation, and **c** H_2_-TPR of the LSC, 73LSCNi-15, and 63LSCNi-15. Reprinted permission from Ref. [107]. **d** Surface exsolution of Ni nanoparticles on the A-site deficient perovskite. **e** Surface exsolution of Ni nanoparticles on the perovskites without defects. Reprinted permission from Ref. [38]

The stoichiometry of perovskite system also has a high effect on the surface exsolution characteristics. Recent studies suggested that the existence of A-site deficiency and oxygen vacancy in perovskite systems could improve the surface exsolution of B-site metals [38, 78, 106]. Neagu et al. [38] addressed the effect of crystal defects on exsolution characteristics in which the perovskite oxides prepared with different defect concentrations were designed and synthesized to investigate the correlation between defect chemistry and surface exsolution. The results showed that an oxygen vacancy concentration higher than ~ 0.05 improves the surface exsolution of nanoparticles reduced under high-temperature (~ 900 °C) processes. The extent of oxygen vacancy formation was improved in the A-site deficient perovskite oxides, La_0.6_Sr_0.3_Cr_0.85_Ni_0.15_O_3 − δ_ (63LSCNi-15), due to their high reducibility compared to stoichiometric perovskite oxides, La_0.7_Sr_0.3_Cr_0.85_Ni_0.15_O_3 − δ_ (73LSCNi-15) and La_0.7_Sr_0.3_CrO_3 − δ_ (LSC), as shown in Fig. 8a–c [107]. This observation is supported by the theoretical and experimental research on cation segregation models inside the perovskite oxide, which demonstrate that the formation of A-site deficiency and the subsequent surface oxygen vacancy improve the nanoparticle exsolution by enhancing the segregation of exsolution cations [60, 108]. The experimental results of Ni nanoparticles also support the theoretical studies. The Ni nanoparticles were highly distributed on the A-site deficient La_0.52_Sr_0.28_Ni_0.06_Ti_0.94_O_3_ perovskite oxide surface, while the A-site stoichiometric sample exhibited almost no surface exsolved nanoparticles [38]. As the level of A-deficiency increased, there was a noticeable enhancement in the extent of exsolved metal on the surface [109]. A similar trend is also demonstrated on the various studies with different perovskite systems [77, 97, 110, 111].

### Crystal Facet

**Fig. 9:**
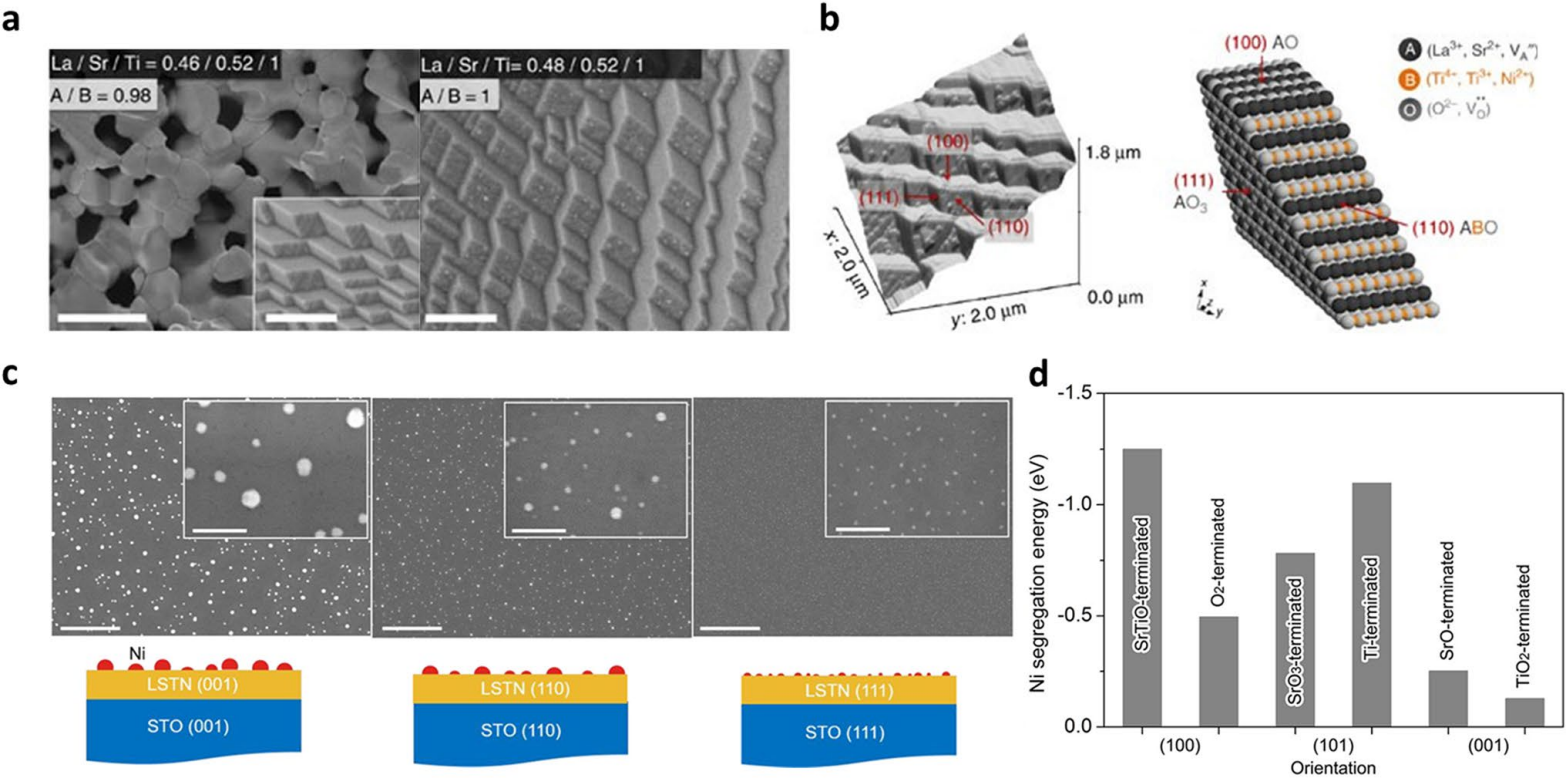
**a** Nanoparticle exsolution on different surface termination. **b** AFM image and atomic scale perovskite model with different surface orientation. Reprinted permission from Ref. [61]. **c** Exsolution of nanometal particles on epitaxially prepared perovskite thin film. Reprinted permission from Ref. [71]. **d** Calculated Ni segregation energy on different surface orientation. Reprinted permission from Ref. [67]

The termination or crystal facet of the perovskite surface also determines the exsolution properties. Figure 9a shows the SEM images of nanoparticle exsolution on different crystal facets [61]. For stoichiometric surface (A/B =  ~ 1), under reducing condition (5% H_2_/Ar, 930 °C, 20 h), the Ni nanoparticles prefer to exsolve on the (110) surface of a (La,Sr)(Ni,Ti)O_3_-based oxide, while the (100) or (111) surfaces exhibit almost no or few exsolution particles. The ABO^4+^/O_2_^4−^ terminated (110) surface is believed to reduce the energy barrier for nanoparticles nucleation, compared to that of A-site terminated (100) or (111) surfaces as illustrated in Fig. 9b. The nanoparticle growth step requires the migration of B-site dopant from perovskite bulk, therefore, the curved trajectory of B-site cation in (110) plane probably reduces the required energy for surface migration of B-site dopant from perovskite bulk to its surface [112]. Also, the A-site deficiency on (110) orientation could lower the repulsion between host A-site cation and B-site dopant, improving the exsolution properties more than other terminations.

Recent studies on the surface termination effect of exsolution utilize epitaxially prepared perovskite thin film samples to precisely investigate its characteristics. Kim et al. [71] prepared the epitaxially deposited La_0.2_Sr_0.7_Ni_0.1_Ti_0.9_O_3 − δ_ perovskite thin film with different surface orientation to characterize the termination effect of exsolution. After reduction in dry H_2_ at 900 °C for 12 h, the nanoparticles exsolved on the (111) surface exhibited the smallest nanoparticle exsolution with homogeneous distribution, compared with that of the (110) and (001) orientation samples (Fig. 9c). This observation originates from the different interfacial energy of each perovskite orientation that influences the nucleation and growth of nanoparticles. A study conducted the reduction (dry H_2_, 900 °C, 24 h) of bulk samples of a A-deficient perovskite oxide, La_0.7_Ca_0.2_Ni_0.25_Ti_0.75_O_3_, and reported similar results, showing a larger population density and smaller size of Ni particles on the (111) surface compared to those on the (100) surface [87]. However, the optimal surface facet for nucleation and growth kinetics can vary depending on the exsolution condition and material properties such as stoichiometry and composition at the surface. This leads to a discrepancy between the native surface and the cleaved surface of synthesized materials due to differences in stoichiometry and surface toughness [38]. To understand the facet dependence of exsolution, it is crucial to conduct comparative experimental and theoretical analyses for each material system under identical conditions. DFT calculations can provide insight into the energetics of cation migration depending on crystal facets in a designed theoretical model during the exsolution process. Gao et al. [67] theoretically studied the segregation energy of Ni cation on different orientations of a SrTiO_3_-based model (Fig. 9c, d). The results showed that Ni nanoparticles were preferentially exsolved on the (100) orientation of perovskite oxide surface. Similar studies on facet dependent exsolution characteristics are also widely conducted with diverse perovskite systems [113, 114].

### Lattice Strain

**Fig. 10:**
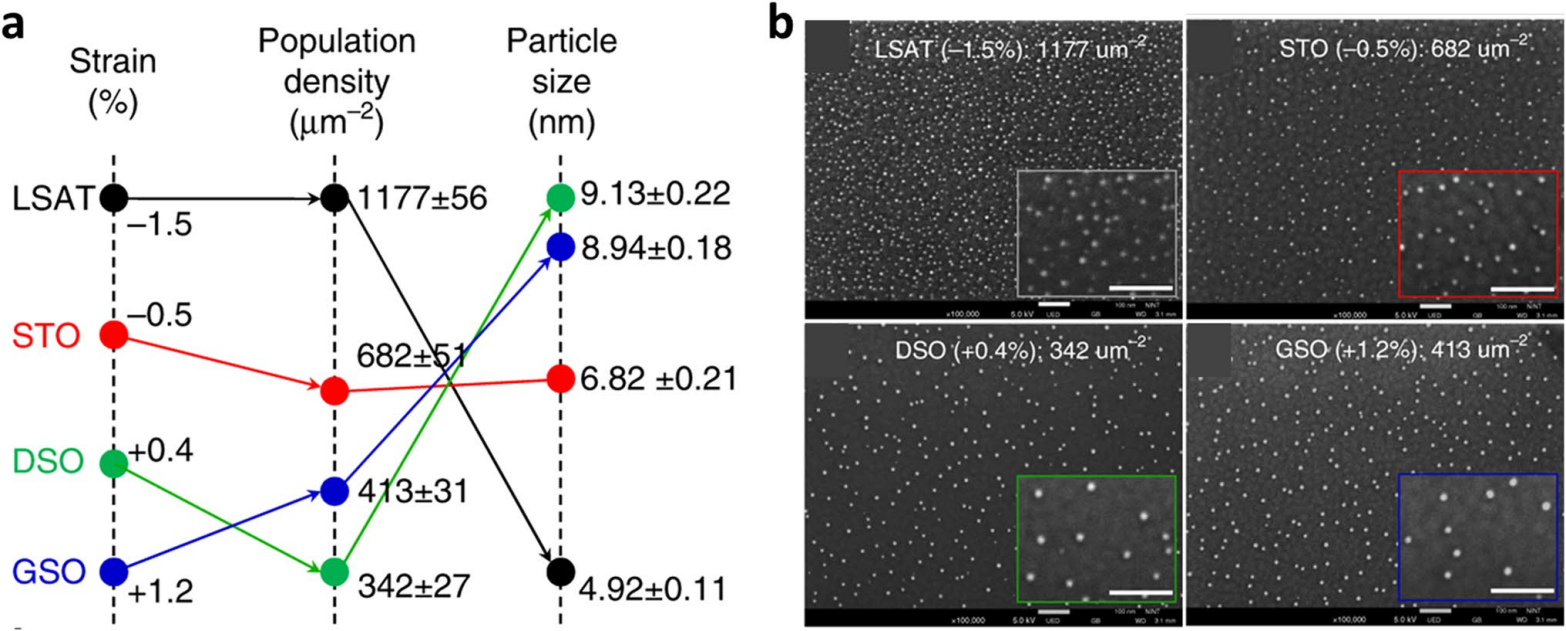
**a** Size and distribution of exsolved nanoparticles as a function of surface strain. **b** SEM images of exsolved nanoparticles prepared on diverse strain-induced perovskite thin films. Reprinted permission from Ref. [115]

The lattice strain of perovskite oxide support could also be a key control factor. Recent studies suggest that the surface strain of perovskite oxide could be regarded as an additional driving force for nanoparticle exsolution, controlling the size and distribution of exsolved nanoparticles. Han et at. [115] introduced the strain-induced exsolution of Ni nanoparticles on the surface of strain-induced perovskite thin films. The epitaxially prepared 100 nm La_0.2_Sr_0.7_Ni_0.1_Ti_0.9_O_3 − δ_ thin film perovskite layers were deposited on the diverse substrates to induce tensile or compressed strain. The results obtained indicated that the exsolved nanoparticles on the compressed strain-induced sample showed a smaller average particle size and higher population density, compared with those of tensile strained samples (Fig. 10a, b). This study explains that the tendency for exsolution originates from the relaxation effect of lattice misfit strain energy (Δ*G*_mr_), which enhances the formation of surface nucleation kinetics for compressive strained samples. Conversely, a contradictory tendency for exsolution was also reported in thin films on substrates with different lattice sizes [77]. In this case, as compared to compressive strain, tensile strain enhances the formation of oxygen vacancies on the La_0.6_Sr_0.4_FeO_3_ (LSF) surface, promoting the Fe^0^ formation. The conducted Monte Carlo (MC) simulations and DFT calculations suggest that oxygen vacancy fairs ($V_{{\text{O}}}^{2 + } - V_{{\text{O}}}^{2 + }$) on the surface play a crucial role in determining nucleation rate rather than isolated oxygen vacancies. Kim et al. [116] investigated the influence of metal-oxide bond strength on the surface exsolution properties by inducing in-plane strain on the epitaxially deposited SrTi_0.75_Co_0.25_O_3 − δ_ (STC) thin film perovskite layers. The weakened Co–O bonding with in-plane tensile strain promoted the surface Co exsolution, suggesting the possible utilization of isovalent doping in exsolution perovskite design to enhance the exsolution properties. Several results insist that possible surface defects and cation-oxygen ion bonding created by lattice strain could be the main control factor that determines the exsolution characteristics for each case. Therefore, we believe that the design of perovskite materials for these studies is also curial in that the inherent structural properties of perovskite would have dominant effect on their strain-induced perovskite thin film systems.

## Unique Properties of Exsolution Catalysts

The deactivation of nanosized catalysts is often attributed to catalyst sintering, carbon deposition, and sulfur poisoning, which pose considerable challenges in maintaining their long-term functionality. Recent studies on exsolution catalysts have shown promising outcomes in terms of enhanced durability with high catalytic activity. As discussed in Sects. 2 and 3, exsolution catalysts are created through a controlled phase separation process under reducing conditions, leading to the formation of metallic nanoparticles embedded on the host matrix. These socketed structures enable strong metal-support interaction, imparting excellent thermal and chemical stability to the nanometal catalysts. Moreover, when the parent perovskite matrix is stoichiometric, the exsolved nanoparticles possess the capability to redissolve into the oxide support lattice under an oxidative atmosphere. These properties are unique to the exsolution phenomenon compared with that of traditional supported nanocatalysts, including infiltration, chemical and physical deposition methods. The following sections demonstrate the unique characteristics of exsolved nanoparticles. The significance of the exsolution process in obtaining these unique properties would be introduced with wide range of applications for heterogeneous catalysis, energy storage, and conversion fields.

### High Thermal Stability and Uniform Distribution

**Fig. 11:**
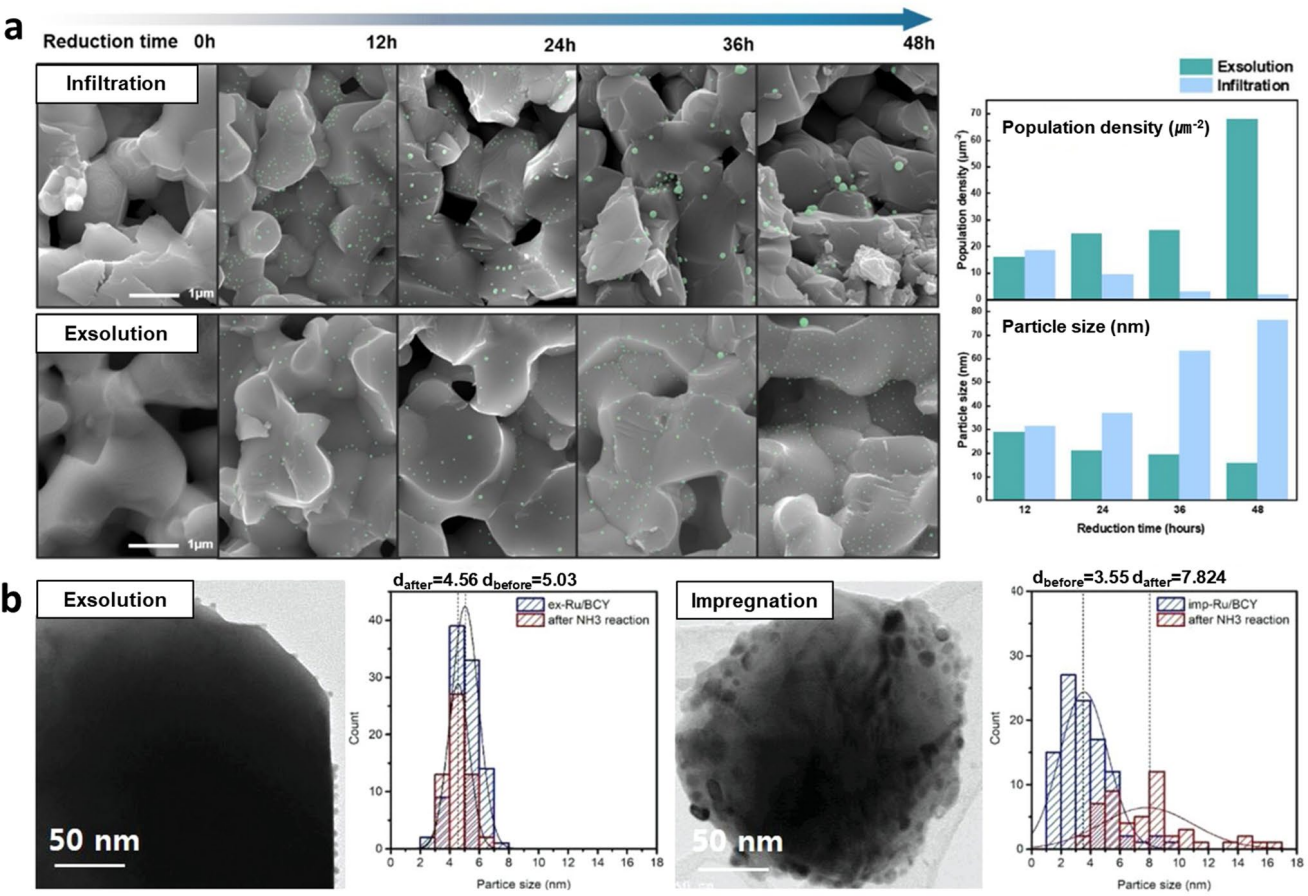
Thermal stability characteristics of exsolved nanocatalysts. **a** Comparison between infiltrated and exsolved Cu nanoparticles in dry H_2_ at 600 °C as a function of reduction time. (Cu particles are highlighted in blue in SEM image). Reprinted permission from Ref. [21]. **b** Comparison between exsolved and impregnated Ru nanoparticles after the ammonia synthesis test. Reprinted permission from Ref. [81]

Exsolved materials exhibit high nanoparticle populations with homogeneous distributions and consistent sizes. These controlled particle distributions can be advantageous for catalytic reactions that require specific particle sizes. The size of the active component is believed to significantly impact on crucial properties, including its durability, catalytic activity and selectivity. Moreover, socketed nanoparticles possess a remarkable ability to resist agglomeration, suggesting that the strong resistance to the undesirable clustering or coalescence of nanoparticles [117]. Han et al. [115] introduced that the exsolved Ni particles exhibited significantly smaller surface diffusion coefficients (4.9 × 10^–23^–8.1 × 10^–23^ m^2^ s^−1^ at 900 °C) compared to conventionally deposited particles (10^–8^–10^–12^ m^2^ s^−1^ at 900–1000 °C). The smaller surface diffusion coefficient of exsolved Ni nanoparticles exhibited the superior anti-agglomeration properties and slower particle coarsening. Neagu et al. [61] examined the changes in the morphology of exsolved and infiltrated Ni particles on (La,Sr)_0.8_TiO_3_-based perovskite oxides before and after heat treatment under 5% H_2_/Ar at 900 °C for 70 h. After aging, the infiltrated particles experienced agglomeration, transitioning from a population density of approximately 35 particles μm^−2^ to approximately 10 particles μm^−2^. On the other hand, ~ 90% of the exsolved particles remained intact with no coalescence, showcasing remarkable resistance to agglomeration. The great resistance to surface particle agglomeration is crucial because particle agglomeration can cause the loss of active surface area of nanoparticles, leading to the critical deactivation of catalytic activity on diverse systems. These unique characteristics make exsolved catalysts highly attractive for a wide range of applications. Figure 11a exhibits experimental observations on the sinter-resistance of exsolved nanoparticles applied to electrode material for solid oxide fuel cells (SOFCs). The exsolved Cu particles exhibited improved catalytic activity compared to catalysts prepared through conventional methods. The Cu nanoparticles prepared via exsolution method demonstrated excellent resistance to aggregation even after long-term operation. The results also showed a higher particle population density and smaller average size of exsolved Cu catalysts compared with the particles prepared by infiltration method. This unique observation is originated from their strong interaction with the substrate [21, 118]. In contrast, infiltrated particles showed severe agglomeration due to weak interaction with the substrate, leading to a larger particle size distribution and an increased particle size. The studies on Ru nanocatalysts also reported similar properties (Fig. 11b). The exsolved Ru nanoparticles prepared on BaCe_0.8_Y_0.2_O_3_ (BCY) perovskite oxide exhibited a great resistance to the particle agglomeration during high-temperature NH_3_ synthesis reaction. Compared to the conventional Ru nanocatalysts prepared via impregnation method, exsolved Ru nanocatalyst showed minor size differences after prolonged operation. The catalytic activity is dependent on the size of catalysts due to the inverse relationship between the size of the nanoparticles and the specific surface area available for the reaction. Consequently, exsolution, which enables precise control over the size of nanoparticles and ensures their sustained small size even during long operations, emerges as a significant technology. The exsolution nanoparticle showed the great thermal and chemical stability for diverse energy system; however, possible coalescing of nanoclusters before socketing remains practical challenge in controlling of the few nanoscale exsolution particles. Therefore, further studies in optimization of its size is required to maximizing the particle distribution.

### Anti-cocking Resistance

**Fig. 12:**
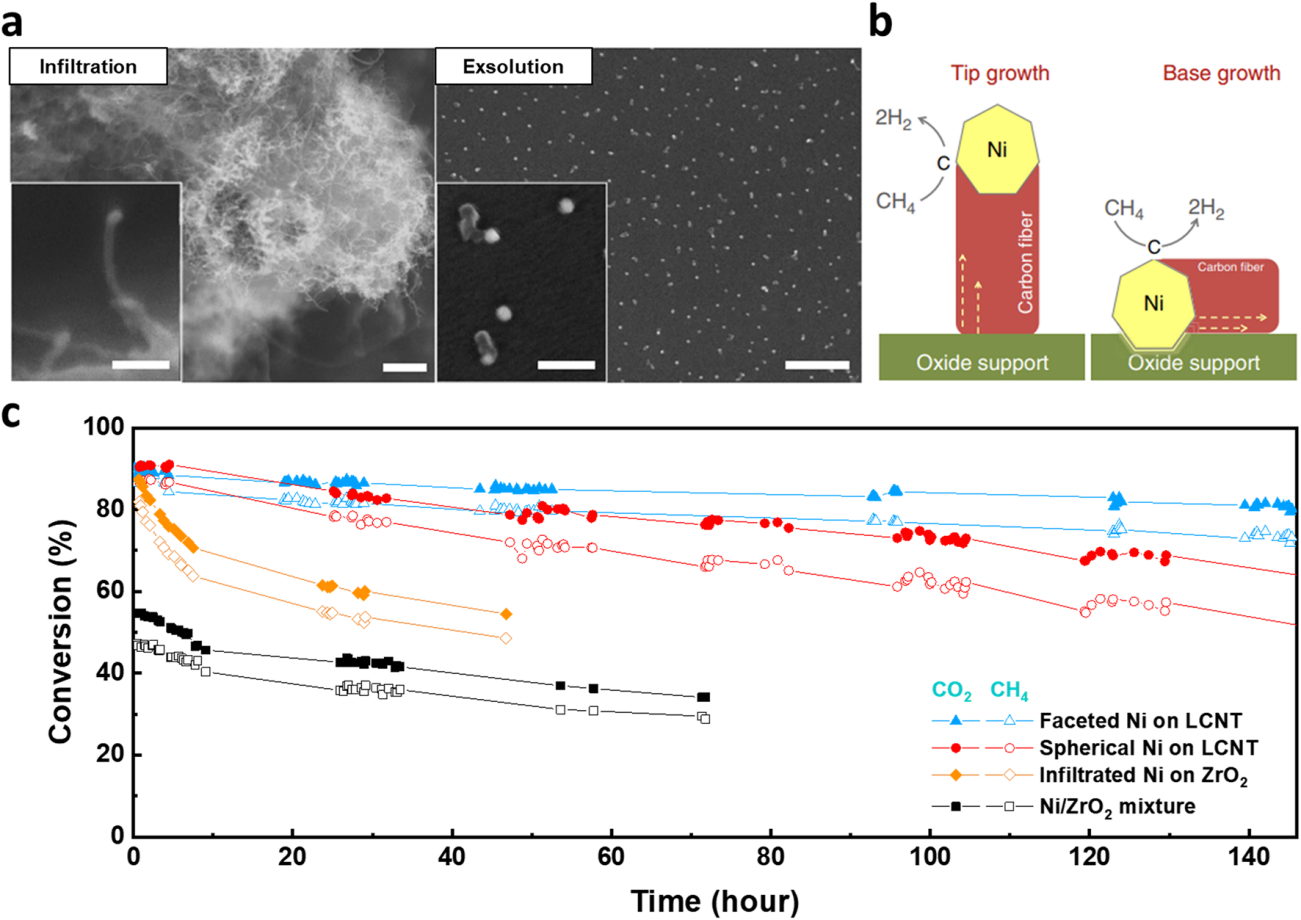
Remarkable anti-coking trait of exsolved catalysts. **a** SEM images of infiltrated and exsolved Ni particles after coking test in 20% CH_4_/H_2_, at 800 °C for 4 h. (scale bars, 0.5 mm (overview); 100 nm (detail)). Reprinted permission from Ref. [61]. **b** Schematic of possible carbon fiber growth mechanisms. **c** Activity of shape-controlled Ni exsolution and reference catalysts in dry reforming of methane at 700 °C (GHSV = 30,000 mL g^−1^ h^−1^). Reprinted permission from Ref. [87]

Recently, there have been many reports highlighting the enhanced coking resistance through the utilization of exsolved Ni nanoparticles in dry reforming of methane (DRM) process. Neagu et al. [38] reported that the exsolved Ni particle systems (La_0.52_Sr_0.28_Ni_0.06_Ti_0.94_O_3 − δ_) exhibited enhanced coking stability and reduced carbon fiber growth compared to the infiltrated and deposited Ni particles (Fig. 12a). Comparing the coking resistance of exsolved Ni particles with infiltrated Ni particles, the different carbon fiber growth mechanism provides insights into the superior performance of exsolved Ni particles (Fig. 12b). The strong interaction between the exsolved particles and the parent oxide support prevented nanoparticle uplifting and subsequent fiber growth without catalyst separation. The carbon coking resistance of the exsolved Ni system is believed to be originated from the superior adhesion properties of exsolved nanocatalyst, which lead to the base growth mechanism of carbon nanofibers. Kim et al. [87] investigated the effect of the shape-control exsolution of Ni nanoparticles in the La_0.7_Ca_0.2_Ni_0.25_Ti_0.75_O_3 − δ_ (LCNT) system on activity and stability in DRM. Remarkably, the faceted Ni catalysts maintained high and continuous conversion activity above 94% for 390 h at 800 °C due to their unique growth mechanism of carbon nanotubes, where the {111} surface prevented surface coverage by carbon cokes. The faceted Ni nanocatalysts prepared via exsolution outperformed the spherical Ni exsolution particles and conventionally infiltrated Ni nanoparticles on ZrO_2_ support (Fig. 12c). A study suggested a carbon cleaning process for the exsolved Ni nanoparticles on the proton conducting perovskite oxide [119]. The hydroxyl and H compounds, which are formed on the perovskite surface from H_2_O molecule play a key role in removing carbon, which leads to the high durability of protonic ceramic fuel cells utilizing various hydrocarbon fuels. As mentioned above, exsolved nanocatalysts demonstrated superior initial performance and prolonged stability compared to the conventional impregnation method. However, an ongoing issue has been the persistent decline in performance over time. Exsolution catalysts exhibit a phenomenon known as “base growth” due to their strong adhesion to the parent phase. Nevertheless, it is worth noting that the active surface area of the catalyst is still compromised due to carbon accumulation. Furthermore, the performance of these catalysts has been found to vary significantly depending on the shape and size of the nanoparticles, indicating a significant influence on the mechanism of carbon growth. To address these limitations and optimize catalyst performance, it is imperative to focus future research efforts on controlling nanoparticle shape and size with precision. Additionally, utilizing methods for regeneration of exsolved catalysts via redox cycles will be instrumental in overcoming the performance degradation posed by carbon cocking, enhancing durability of these catalysts. The following section focuses on the self-regeneration of exsolution catalysts.

### Self-Regeneration in Redox Conditions

**Fig. 13:**
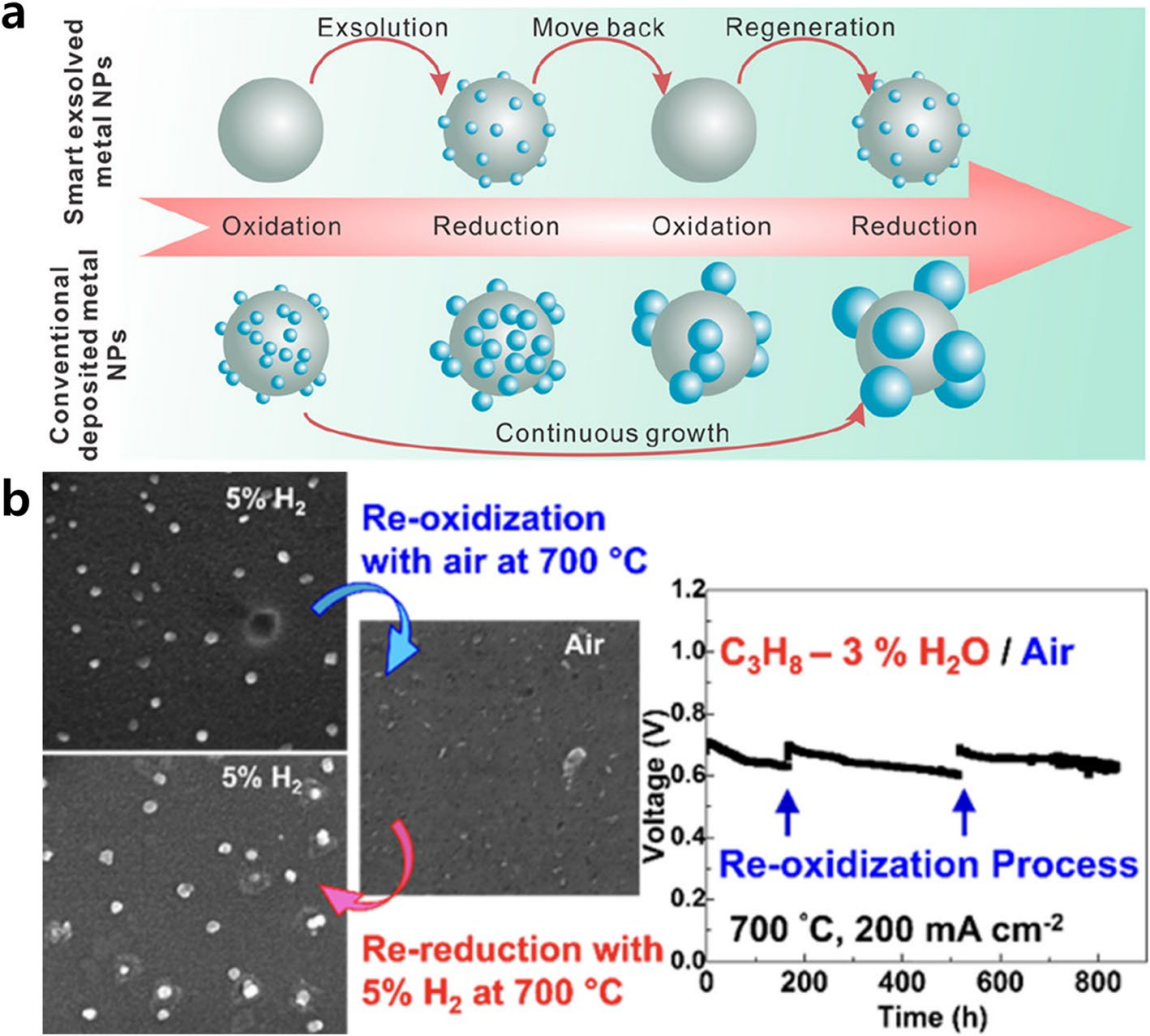
Regenerative capability of exsolved particles. **a** Schematic illustration of self-regeneration mechanism. Reprinted permission from Ref. [55]. **b** The redox reversibility and stable long-term performance of a perovskite anode material, La_0.3_Sr_0.7_Cr_0.3_Fe_0.6_Co_0.1_O_3 − δ_ with exsolved Co–Fe nanoparticles. Reprinted permission from Ref. [121]

The exsolution catalyst exhibits a notable feature of self-regeneration in redox environments. The occurrence of reversible or irreversible exsolution in perovskite-based systems is primarily determined by the A-site stoichiometry. Reversible exsolution is mainly observed in stoichiometric perovskites (ABO_3_), while irreversible exsolution occurs in A-site-deficient perovskites (A_1 − α_BO_3_) [45, 120]. In the previous sections, the concept of irreversible exsolution, wherein nanoparticles are strongly embedded on perovskite substrates, is explored as a strategy to enhance stability by mitigating aggregation and carbon deposition. Reversible exsolution allows for periodic redox cycle, promoting catalyst regeneration, preventing agglomeration, and greatly extending their longevity. Figure 13a illustrates the self-regeneration capability of exsolved nanoparticles in a redox environment compared to conventionally prepared catalysts, which undergo continuous particle growth through coalescence during prolonged exposure to elevated temperatures [55]. Self-regenerative exsolved nanoparticles possess the capability to return to the oxide support when subjected to high pO_2_ (oxidative atmosphere) treatment and can be regenerated through reduction, primarily occurring in the vicinity of the surface area where an intermediate phase may be present. Lai et al. [121] demonstrated the promising possibility of the exsolved Co-Fe alloy in the La_0.3_Sr_0.7_Cr_0.3_Fe_0.6_Co_0.1_O_3 − δ_ perovskite system as a self-regeneratable SOFC anode material with excellent redox reversibility, and stable performance for over 800 h with propane at 700 °C (Fig. 13b). Although a complete recovery of the initial oxidation state was not reported, the initial nanoparticles and performance were recovered even though the dissolved metal was only partially recombined. Self-regeneration of exsolved nanoparticles can be utilized by repeating redox cycling, which alleviates the problem of sintering and coking of supported nanocatalysts.

## Novel Strategies for Improving Exsolution

Conventionally, gas reductions using hydrogen have been employed to induce the exsolution of nanoparticles on an electrode. However, this method encounters significant limitations such as the sluggish cation diffusion rate, instability of the host oxide, and insufficient thermodynamic driving force. Numerous studies have developed new approaches to overcome the aforementioned limitations. For example, several strategies were developed to enhance the kinetics of nucleation and growth through the high thermal, photonic, or electrical energy-driven exsolution. These strategies open up opportunities to broaden the application of exsolved materials. The subsequent sections provide an overview of these strategies and offer insights into discovering new approaches to enhance exsolution behaviors.

### Electrochemical Switching

**Fig. 14:**
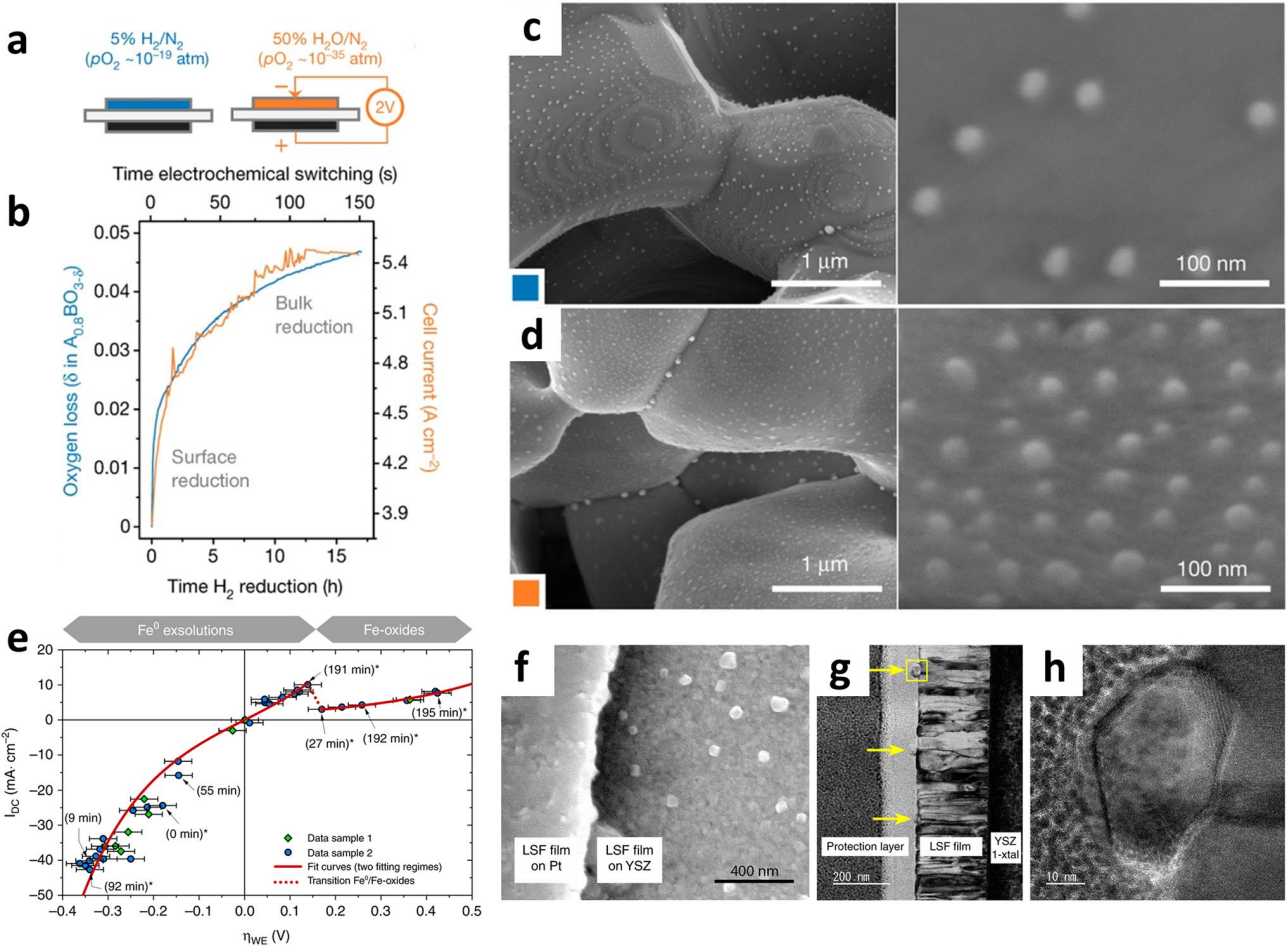
Electrochemical switching to trigger exsolution on the perovskite oxide. **a** Reduction conditions and **b** reduction tendencies of fuel electrodes using gas reduction and electrochemical switching methods. Morphologies of electrode surface after **c** traditional H_2_ reduction for 20 h and **d** electrochemical switching process (2 V, 150 s). Reprinted permission from Ref. [1]. **e** Plot of the area-normalized current (*I*_DC_) versus overpotential dropping at the LSF thin film working electrode. **f–h** Electron microscopic images of exsolved Fe nanoparticles on the LSF thin film after 60 h under reduction conditions. Reprinted permission from Ref. [123]

Applied potential in the electrochemical device induces reactions and the migration of ions, which often leads to electrocatalytic activation. Myung et al. reported that the exsolution behavior can be optimized by applying a potential to solid oxide cells without the presence of reducing gases such as H_2_ [1]. The electrochemical switching strategy presented notable advantages over hydrogen gas reduction, as evidenced by a shorter completion time (150 s compared to 17 h) and significantly higher population density of nanoparticles formed on a La_0.43_Ca_0.37_Ni_0.06_Ti_0.94_O_3 − δ_ fuel electrode at a potential of 2 V in a 50% H_2_O/N_2_ environment (Fig. 14a–d). As a result, there was a significant increase in peak power density after electrochemical switching, accompanied by a twofold rise in population density compared to gas reduction. The estimated pO_2_ under an applied potential of 2 V is 10^–35^ atm according to the Nernst equation, which is extremely lower than the 3%H_2_O/H_2_ environment (10^–19^ atm), thereby promoting the exsolution process. This method has been used in the field of exsolution research. In their respective studies, Kim et al. and Jo et al. reported that electrochemical reduction played a crucial role in promoting the exsolution of metal nanoparticles by mitigating cation diffusion limitations and facilitating nucleation [21, 90]. Other studies have demonstrated the use of electrochemical switching under various environmental conditions to drastically accelerate the exsolution process [64, 122]. The voltage-driven process accelerated the kinetics and leads to efficient exsolution, resulting in enhanced catalytic performance.

To explore reversible electrochemical switching, Opitz et al. investigated the redox exsolution on a La_0.6_Sr_0.4_FeO_3 − δ_ (LSF) thin film for fuel electrodes, which was deposited by pulsed laser deposition (PLD), by changing the applied electrochemical voltage under H_2_O/H_2_ environments [123]. Figure 14e–h presents the observation of voltage-driven Fe^0^ exsolution under anodic polarization and a phase transition to Fe-oxides (Fe_1 − *x*_O and Fe_3_O_4_) relating to the non-linear I–V region (+ 150 ± 46 mV) under cathodic polarization. This result demonstrates reversible control of the metal and oxide phases of exsolved particles by adjusting the electrochemical potential. It is worth noting that exsolved particles are not completely re-incorporated into the host lattice under cathodic polarization. Zhou et al. [41] reported that Ag nanoparticles were exsolved under cathodic polarization through deintercalation in the (La_0.8_Sr_0.2_)_0.95_Ag_0.05_MnO_3 − δ_ for air electrodes. The exsolved Ag on the surface considerably improved the catalytic activity of the oxygen reduction reaction. Under anodic polarization, Ag re-intercalated into the perovskite lattice, allowing for easy restoration of the electrode without damage.

### High Energy Exsolution

**Fig. 15:**
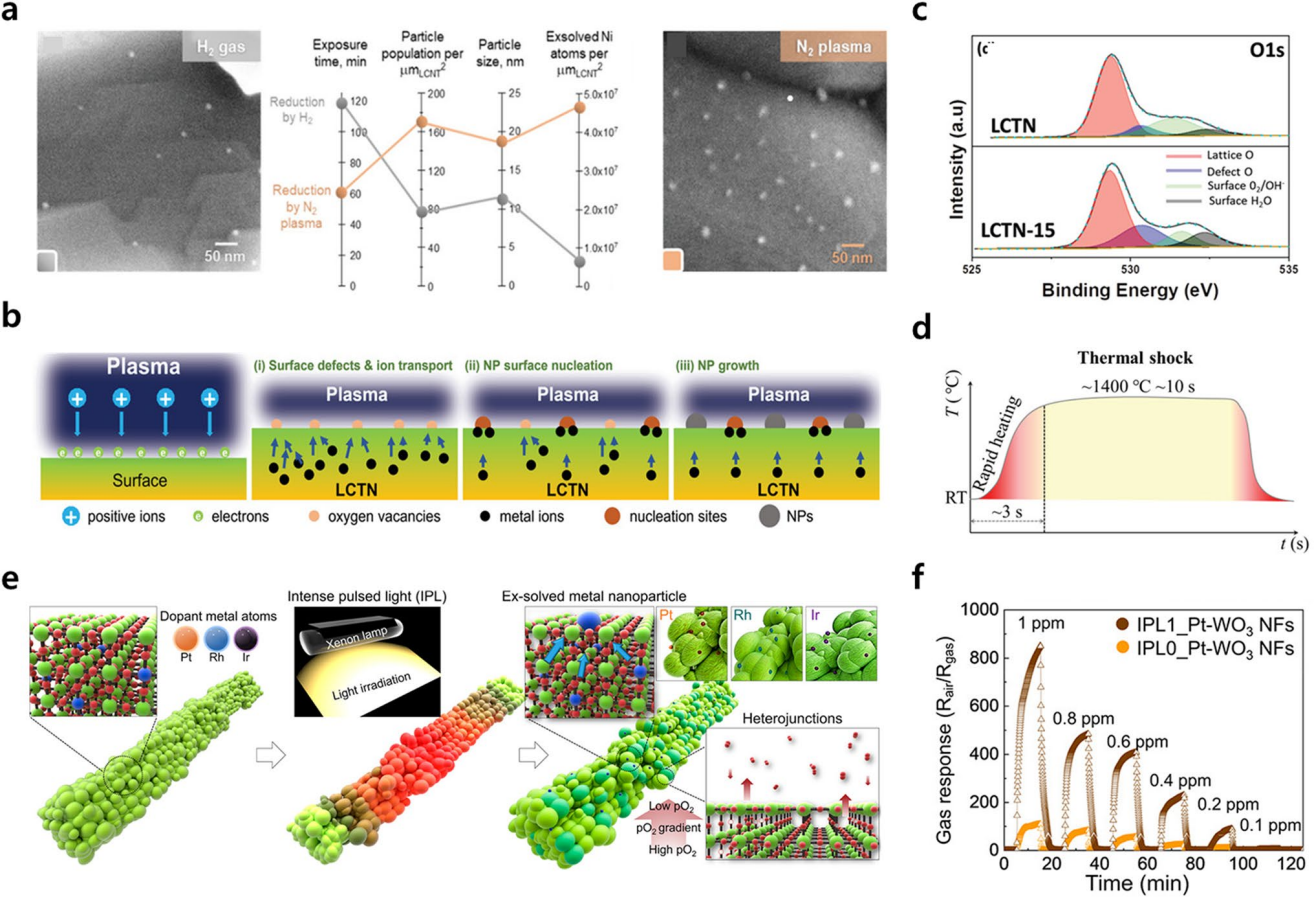
**a** SEM and characteristics analysis of the Ni nanoparticles grown on the La_0.43_Ca_0.37_Ni_0.06_Ti_0.94_O_3 − d_ perovskite surface after exposure to H_2_ gas for 2 h (gray) and N_2_ plasma for 1 h (orange) at 650 °C. Reprinted permission from Ref. [86]. **b** Schematic representation of plasma-driven exsolution of Ni nanoparticles indicating various phenomena occurring during the process. **c** XPS results of O 1*s* of LCNT before (LCNT) and after (LCNT-15) Ar plasma treatment. Reprinted permission from Ref. [125]. **d** Temperature profile for the thermal shock technique. Reprinted permission from Ref. [126]. **e** Schematic diagram of exsolved noble metals decoration on WO_3_ NFs via IPL-MP treatment with a mechanism of heterojunctions and exsolution formation. **f** Hydrogen sulfide (H_2_S) gas sensing results from 1 to 100 ppb concentration of IPL exsolved Pt–WO_3_ NFs. Reprinted permission from Ref. [127]

Researchers have explored universally applicable exsolution maximization strategies for other applications including sensors or reforming catalysts. Recently, there have been many reports of achieving exsolution in a short time using high energy sources such as thermal shock and plasma as a driving force. Vasileios et al. [86] introduced N_2_ plasma as a triggering agent for exsolution on La_0.43_Ca_0.37_Ni_0.06_Ti_0.94_O_3 − d_. When exposing to N_2_ plasma treatment for 1 h at 650 °C, the exsolved nanoparticles exhibited a higher particle population and size of 170 particles μm^−2^ and 18 ± 1 nm compared to those produced by conventional H_2_ reduction (80 particles μm^−2^, 11 ± 1 nm) (Fig. 15a). This demonstrates that the plasma for exsolution had a stronger effect on nucleation and growth kinetics than the gaseous reducing agent. In the plasma-driven exsolution, the ion and electron fluxes strike and recombine on the oxide surface, creating a dynamic electron layer that promotes nucleation at more points across the surface (Fig. 15b) [124]. Moreover, plasma exsolution was demonstrated at room temperature using an Ar plasma. Khalid et al. [125] exposed LCNT of the same composition to Ar plasma for 15 min at room temperature, and the population density of exsolved Ni nanoparticles was 550 particles μm^−2^ with an average size of 19.7 nm. Based on the plasma sheath theory, it is expected that the flux of electrons in Ar plasma on the LCNT surface is increased more than 100 times compared to that when N_2_ plasma is used, resulting in more nucleation sites. In addition, the XPS analysis confirmed that Ar ions caused sputtering to a depth of 2 nm on the perovskite surface and formed oxygen vacancies (Fig. 15c). It was calculated through DFT that an energy of 4.84 eV was required to form an oxygen vacancy in CaTiO_3_, and 5.38 eV was required when Ni was doped. The energy of the Ar plasma was 10–40 eV, which is sufficient to form oxygen vacancies. Consequently, the combination of oxygen vacancies and a dynamic electron layer facilitated the nucleation and growth of Ni from perovskite oxide, and achieved high density Ni nanoparticles at room temperature within 15 min.

Sun et al. [126] suggested a thermal shock method using carbon paper that leads to an extremely fast increase in the temperature (> 1400 °C), which acts as a triggering agent for exsolution. From the thermodynamic and kinetic perspectives, the exsolution process can be triggered by the thermal potential acting as the driving force. In this case, if the driving force supplied to the host perovskite exceeds the energy barrier of the exsolution, the exsolution kinetics would be greatly enhanced and dynamically occur. The carbon paper was used as a heating source with a heating rate of ~ 10^4^ °C min^−1^ and the perovskite oxide was thermally shocked at 1400 °C for ~ 10 s in 5%H_2_/N_2_ atmosphere (Fig. 15d). The thermal shock-induced exsolutions showed a particle population of 61 particles μm^−2^ with an average size of 14 ± 3 nm, which was higher than the conventional chemical gas-induced exsolution (5%H_2_/N_2_ at 900 °C for 20 h). The extraordinary driving force supplied by thermal shock promoted nucleation sites, and a short treatment time and an increase in the number of nucleation sites, leading to an enhanced concentration of active sites for electrochemical reactions. To evaluate the electrochemical performance of the exsolution perovskite, different kinds of electrolyte-supported solid oxide cells were prepared: pristine, chemical gas-induced, and thermal shock-induced exsolution. The thermal shock-induced exsolution cells showed the maximum power density of 1 W cm^−2^ with a polarization resistance of 1.5 Ω cm^2^ at 900 °C, which exhibited 12-fold and fourfold higher performance compared to pristine cell (0.09 W cm^−2^, 8.3 Ω cm^2^) and chemical gas-induced exsolution cell (0.28 W cm^−2^, 3.8 Ω cm^2^), respectively.

In addition, exsolution through thermal energy has been implemented in non-perovskite oxides, which could not be previously used as hosts because of the strong reduction conditions of the exsolution. Shin et al. [127] introduced intense pulsed light-derived momentary photothermal (IPL-MP) treatment as a triggering agent for exsolution of the Pt, Ru, and Ir nanoparticles from the WO_3_ nanofibers (NFs). As the IPL beyond the bandgap of WO_3_ (2.4–2.8 eV) was irradiated to Pt, Ru, and Ir doped WO_3_, the temperature was rapidly increased above 1000 °C by momentary photothermal with a heating rate of ≈ 4 × 10^5^ °C s^−1^. Elevated temperatures (> 1000 °C) provide a sufficient driving force for the release of oxygen atoms from the oxide lattice. This release formed an oxygen concentration gradient at the interface between the oxide lattice and the ambient air, creating a quasi-reducing environment that further supports the release of oxygen atoms. Consequently, the samples treated by IPL underwent a partial reduction of the oxide lattice near the surface, leading to the creation of multiple WO_3 − *x*_/WO_3_ heterojunctions as well as exsolved nanoparticles (Fig. 15e). These combined effects result in elevated catalytic activity and enhanced chemiresistive sensitivity. In particular, IPL-treated Pt doped WO_3_ NFs exhibited an extraordinary gas response (*R*_air_/*R*_gas_) of 852.4 at 1 ppm of H_2_S exposure, which is 7.9-fold higher than that of Pt doped WO_3_ NFs before IPL-MP treatments (Fig. 15f). In addition, it exhibited outstanding selectivity toward H_2_S against other interfering gases, including 1 ppm of NH_3_, C_2_H_5_OH, C_3_H_6_O, and C_2_H_6_S.

### Phase Transition

The phase transition engineering of perovskite materials has been developed as one of the promising strategies to facilitate exsolution [128]. Under various intrinsic and extrinsic conditions, the perovskite materials underwent phase transition into relate phases such as double (AA′B_2_O_5+δ_) or Ruddlesden-Popper (RP, A_2_BO_4_) perovskite phases, accompanying the exsolution of metallic nanoparticles [129–131]. These perovskite-related structures exhibit high electrical conductivity and rapid oxygen ion diffusion due to their unique oxygen vacancy channel and structural characteristics [132–134]. Furthermore, the redox-reversible properties accompanied by the self-regeneration of nanoparticles of these materials have been proposed as crucial attributes for enhancing electrocatalytic performance [89, 102, 129]. While these perovskite materials exhibit remarkable exsolution properties and catalytic activities, they seemingly lack the crystal structural stability compared to single perovskite materials [135]. Recently, several studies have focused on maximizing the exsolution through the phase transition properties of perovskite materials, while also restraining crystal structure decomposition to enhance the stability of these materials [136].

**Fig. 16:**
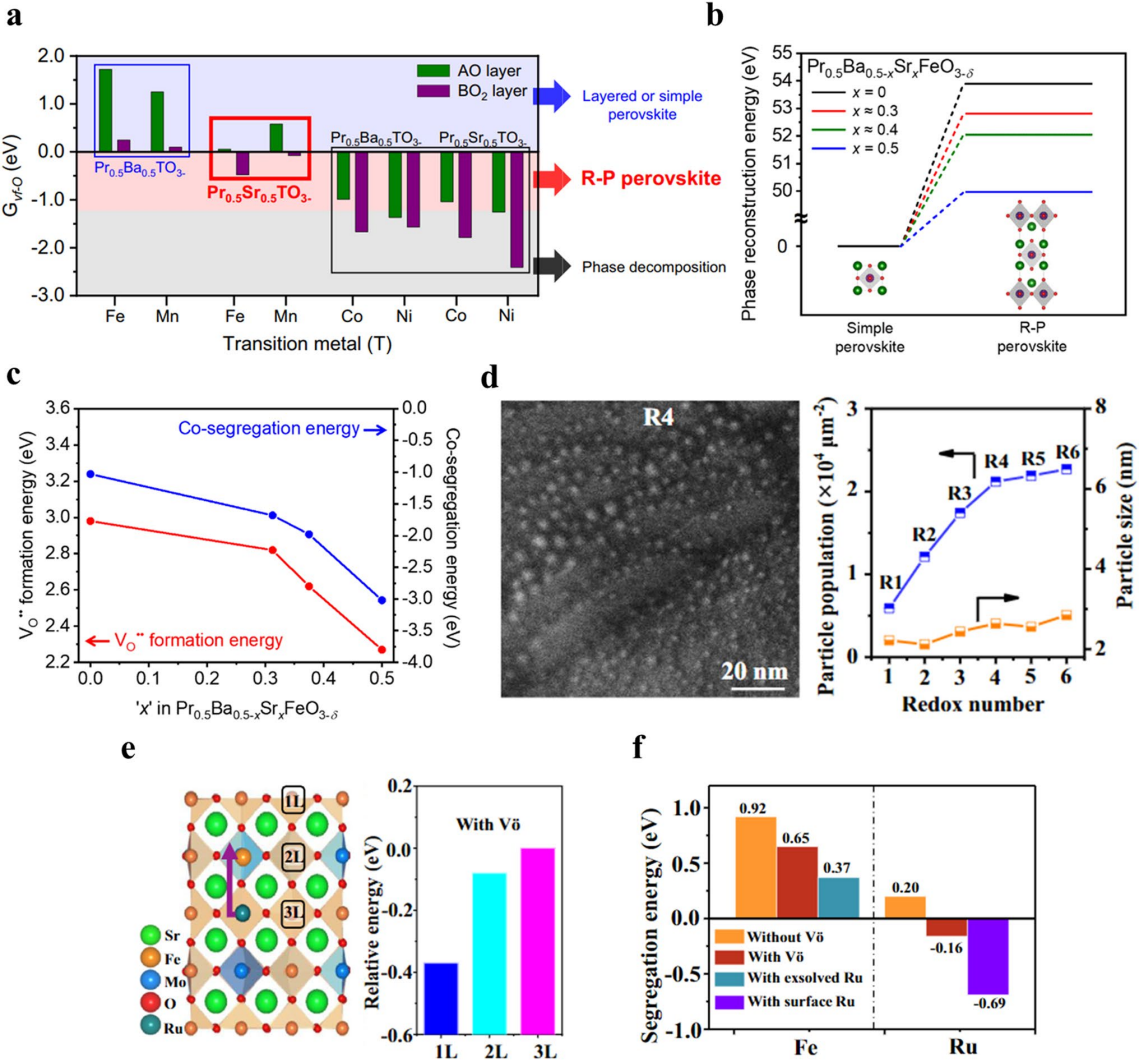
Exsolution and phase transition properties in perovskite systems. **a** DFT calculated profiles of Gibbs free energy for oxygen vacancy formation from the surface AO (A-site) and BO_2_ (B-site) networks. **b** Total phase transition energy from single to RP perovskite. **c** Oxygen vacancy formation and co-segregation energies as function of Sr doping ratio. Reprinted permission from Ref. [137]. **d** SE-STEM images of SFRuM after 4.^th^ repeated phase transition and population and size of exsolved nanoparticles as a function of redox number. **e** Segregation energies of Fe and Ru in diverse conditions. **f** Relative energy of the slabs as a function of Ru position. Reprinted permission from Ref. [89]

Kim et al. [137] investigated the phase transition characteristics in single perovskite system under reducing environment. To determine the phase transition, the Gibbs free energy for oxygen vacancy formation (G_vf-O_) was calculated for Pr_0.5_Ba_0.5_TO_3 − δ_ and Pr_0.5_Sr_0.5_TO_3 − δ_ (T = Mn, Fe, Co, and Ni) single perovskite systems. DFT calculation results indicated that the G_vf-O_ values should be in a desirable range depending on the lattice layer (A-site G_vf-O_ > 0 eV and − 1.2 eV < B-site G_vf-O_ < 0 eV) for phase transition to RP perovskite without phase decomposition (Fig. 16a). Accordingly, the Pr_0.5_Ba_0.5−x_Sr_x_FeO_3−δ_ system was determined as one of the possible candidates for the complete phase transition to RP perovskite. The role of the Sr doping ratio in the phase transition to RP structure was revealed using DFT calculations. (Fig. 16b, c). The total energies for the phase transition decrease with increasing Sr doping ratio, indicating that the incorporation of Sr^2+^ into A-site facilitates the phase transition to RP structure (Fig. 16b). Furthermore, the increasing Sr doping effectively reduces oxygen vacancy formation energies and co-segregation energies, suggesting the facilitated formation of oxygen vacancies and an enhanced degree of Fe exsolution (Fig. 16c).

There have been previous efforts to facilitate the exsolution of metal nanoparticles using phase transformation engineering [101, 138]. Wang et al.[89] reported that the repeated redox manipulation in reversible phase transition system facilitated the exsolution of Ru, Fe to the surface. Sr_2_Fe_1.4_Ru_0.1_Mo_0.5_O_6 − δ_ (SFRuM) double perovskite system exhibited a reversible phase transition during repeated redox manipulation. Upon reduction (SFRuM R1), SFRuMR1 sample displayed a mixture of RuFe alloy phase and the RP perovskite, and it reconstructed back to its initial state after re-oxidation (SFRuM O1). The repeated phase transitions resulted in enrichment of dopant Ru cations underneath the perovskite surface, facilitating the exsolution of RuFe alloy nanoparticles (Fig. 16d). Figure 16e shows the calculated co-segregation energies of Ru and Fe under different conditions. This calculation revealed that the surface enrichment of Ru decreased the co-segregation energy of Ru with V_o_ from − 0.16 to − 0.69 eV. Furthermore, the Ru exsolution contributed to a decrease in the segregation energy of Fe to 0.37 eV, further promoting the exsolution of the RuFe alloy. Figure 16f displays the calculated total energies of SFRuM Slabs with Ru positions. The surface-segregated state (1L) was more stable than the solid solution one (L3), indicating that the exsolution may be attributed to the preferential migration of Ru, leading to the formation of Ru metal nanoparticles. The dynamic phase transition process was found to enhance the exsolution of nanoparticles in perovskite materials, consequently improving their catalytic properties for a range of electrochemical applications [139, 140]. Research aimed at enhancing the stability and long-term performance of perovskite materials through phase transition is still relatively limited. If further research into this matter becomes more continuous, these materials are expected to play a significant role in the field of exsolution materials.

### Bimetal Exsolution

**Fig. 17:**
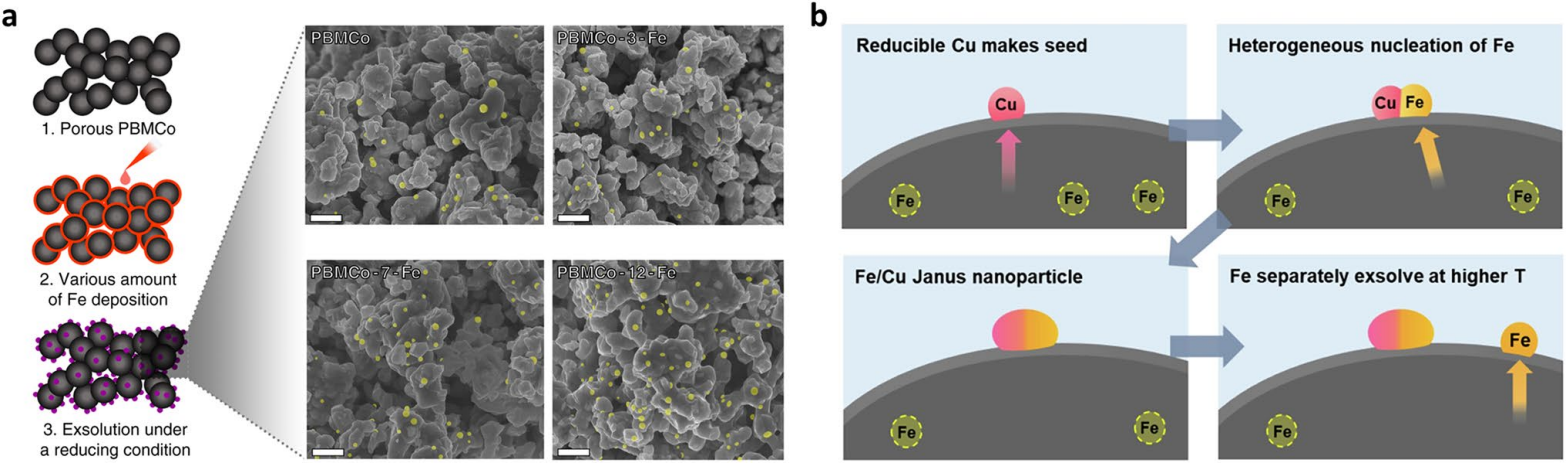
Bimetal exsolution strategies for triggering nanoparticles formation. **a** Schematic illustration and surface morphologies of Co exsolution with topotactic ionic exchange method. Reprinted permission from Ref. [135]. **b** Schematic illustration of seeded effect for Cu–Fe nanoparticle exsolution. Reprinted permission from Ref. [64]

Recent studies on bimetal exsolution suggest the possible utilization of bimetal system for maximizing exsolution properties. Some transition metal cations could be triggered to exsolve on the perovskite surfaces by surface modification processes, including topotactic ionic exchange and seeded effect. The topotactic ionic exchange process takes place by exchanging the positions of the perovskite host cation and the guest cation deposited on the surface. The segregation energy difference between host and guest cation is regarded as the key driving force that triggers the exsolution. Joo et at. [135] introduced the triggered Co exsolution on PrBaMn_1.7_Co_0.3_O_5 − δ_ (PBMCo) layered perovskite system by cation exchange between surface deposited Fe ions and host Co ions. The surface segregation energies of Co and Fe were calculated to be − 0.55 and − 0.15 eV, respectively. The lower surface segregation energy of Co is exsolved on the PBMCo perovskite surface, while the higher segregation energy of surface Fe ions is doped inside the PBMCo perovskite lattice site and maintains its structure. The experimental tendency also supports the theoretical result that a higher surface concentration of Fe ions triggers more surface formation of Co nanoparticles (Fig. 17a). Similar studies on exsolution with topotactic ionic exchange showed similar observations [141, 142].

The seeded effect mechanism is also regarded as an innovative method to trigger nanoparticle exsolution. Compared with the topotactic ionic exchange process, the seeded effect only triggers the exsolution of doped cations without ionic exchange between host and guest as illustrated in Fig. 17b. Jo et al. [64] reported the triggered Fe exsolution from the La_0.43_Sr_0.37_Fe_0.12 − *x*_Cu_x_Ti_0.88_O_3 − δ_ perovskite system. The experimental results showed that the Fe ions were exsolved at the nucleation point of the first exsolved Cu nanoparticles, resulting in the formation of Janus structured Cu–Fe nanoparticle exsolution. The surface infiltrated Cu or Co ions on Fe doped La_0.43_Sr_0.37_Fe_0.12_Ti_0.88_O_3 − δ_ also exhibited a similar triggering effect for Fe exsolution. This observation originates from the reduction in interface formation energy for nanoparticle nucleation, where the pre-existing surface Cu or Co ions provide the metal-oxide interface region for additional Fe exsolution.

### Features of Exsolution Strategies

In the previous sections, we presented a multitude of studies focused on various strategies to enhance exsolution behaviors. In this section, we have summarized the characteristics of each of these novel strategies in terms of benefits and drawbacks, as illustrated in Table 2. Electrochemical switching is a powerful technique for quantitatively controlling pO_2_ conditions in electrodes by adjusting potential in a cell. The controlled pO_2_ can result in exsolution and re-oxidation. Maximizing exsolution kinetics by a voltage-driven process leads to higher population density and larger exsolved atoms for shorter process times compared to gas reduction processes; however, this reduction method has restrictions mainly on electrochemical cells due to its structural limitations. At this point, plasma exsolution has been proposed as a versatile method applicable to various fields, including sensors and reforming catalysts. It demonstrates rapid exsolution kinetics and high population densities by simply exposing the host oxide to plasma. Nevertheless, it should be noted that this approach requires an expensive RF power generator for the plasma and an additional plasma exposure step. Thermal shock can induce exsolution within an incredibly brief timeframe (~ 10 s). This approach does not necessitate a reducing atmosphere. Therefore, materials that are typically unstable in a reducing atmosphere can be utilized as host oxides for exsolution in this process. Nonetheless, it is essential for the oxide host to possess a high level of thermomechanical stability as it needs to endure rapid thermal shocks at elevated heating rates (~ 105 °C s^−1^).

**Table 2:** Benefits and drawbacks of novel approaches in exsolution processes

Exsolution strategies	Benefits	Drawbacks
Electrochemical switching	-Remarkably short exsolution time (~ 150 s) -Enhancement of exsolution kinetics -Large population density -Large amount of exsolved atoms -pO_2_ in electrode can be controlled by electrochemical potential	-Need to fabricate electrochemical cells
Phase transition	-Driving force for triggering exsolution -Boosting exsolution through redox manipulation -Large population density -Simple in situ process	-Lack of lattice structure stability
High energy exsolution		
Plasma	-Short exsolution time (~ 60 min) -Enhancement of exsolution kinetics -Large population density -Large amount of exsolved atoms	-Need to use expensive equipment (RF power source)
Thermal shock	-Remarkably short exsolution time (~ 10 s) -High heating rate (> 50 °C min^−1^)	-High thermal stability of host materials required
Bimetal exsolution		
Topotactic ion exchange	-Driving force for improving exsolution kinetics -Enhancement of lattice structure stability -Large population density	-Additional processes required (infiltration or ALD)
Seeded effect	-Driving force for lowering exsolution temperature -Simple in situ process	-Need to synthesize co-doped oxide materials

Phase transition and bimetal exsolution methods play a key role in triggering exsolution and improving nucleation kinetics. The phase transition in perovskite materials is one of the powerful driving forces promoting the exsolution of metal nanoparticles, allowing straightforward in situ exsolution under SOFC operating conditions with no requirement for additional processes. Unfortunately, excessive phase flexibility can lead to the deconstruction of the crystal structure and the unexpected formation of insulating phases. The topotactic ion exchange method facilitates exsolution, particularly in nucleation kinetics, while maintaining structural stability by changing the position of the deposited metal and host cations. However, achieving topotactic ion exchange during exsolution may require additional processes such as infiltration and atomic layer deposition (ALD). On the other hand, the seeded effect-driven exsolution can be achieved through relatively simple co-doping and reduction processes. During the reduction process, highly reducible metals are preferentially exsolved on the surface and serve as seeds for triggering the exsolution of other metals with relatively low reducibility, resulting in lowering the exsolution temperature. These bimetal exsolution processes lead to the formation of alloy exsolution particles, which can exhibit synergy effects in various applications.

In summary, there are two main approaches to enhance exsolution kinetics: (1) Electrochemical switching, plasma-driven exsolution, and thermal shock methods improve exsolution kinetics through replacement with reduction processes. (2) Phase transition, topotactic ion exchange, and seeded effects utilize chemical and physical effects during reduction processes to obtain the driving forces for exsolution. It is worth noting that these two groups of methods can be used in combination, and this synergy is expected to maximize exsolution behaviors.

## Conclusion and Future Prospects

Over the course of more than a decade, numerous studies have investigated the exsolution method. Most studies have placed a primary emphasis on applications, particularly in the field of energy conversion. Throughout these various studies, significant efforts have also been made to understand the mechanisms controlling and underlying exsolution phenomena. In this review, we have combined the revealed facts to explore the driving forces, processes, controlling factors, unique functionalities, and novel approaches to the exsolution phenomenon. Understanding the underlying mechanism offers valuable insights into the control of exsolution particles and the comprehension of their distinctive characteristics. The unique nature of exsolved particles plays a crucial role in achieving enhanced catalytic activity and durability, thereby expanding the range of their utilization [143, 144]. Currently, developed strategies to enhance the behaviors of exsolved materials have improved utility, time efficiency and cost-effectiveness. Nevertheless, there is still a need for a theoretical and mechanistic understanding to achieve quantitative control of exsolution. For example, the precise control of composition in alloy exsolutions presents a significant challenge due to a lack of understanding of the kinetics in the exsolution of individual metals. The following sections focus on offering perspectives and strategies to address these limitations in understanding exsolution phenomena.

### Fundamental Mechanism and Control Factors

While the thermodynamics of exsolution, including driving forces, has been extensively explored, a fundamental understanding of the kinetics of nucleation and growth of exsolution particles is still lacking. Although several recent studies have utilized theoretical modeling to gain insights into the behavior of exsolved nanoparticles such as the quantity of exsolution and particle size, accurately controlling and predicting the exsolution phenomenon remains challenging. To effectively conduct mechanistic analyses, it is crucial to establish clear sample and condition settings, considering practical effects arising from factors like crystal facets, impurities, grain size, and boundaries, as discussed in this review. In addition, the exsolution phenomenon is highly intricate due to the various processes and interactions occurring between the particles and the supporting material. Thus, variables such as temperature can affect several factors, including particle size, population, and shape. To address these limitations, it is necessary to conduct modeling studies that integrate various factors into each process.

Understanding the nucleation process poses significant challenges across various fields of study in practice due to the inherent difficulty in capturing nucleation events [75]. Fortunately, recent studies have successfully observed the nucleation and crystallization of exsolution particles from the mother phase using in situ TEM [59, 78, 79]. These findings highlight the potential of exsolvable materials as promising samples for capturing nucleation. Although instances of such cases are uncommon, we believe that further theoretical and experimental investigations can incrementally enhance the understanding of nucleation dynamics in phase separation.

### Shape-Control

Faceted nanoparticles have emerged as active and selective catalysts due to their high surface-area-to-volume ratios, numerous edge/kink sites, and desired surface facet, which exhibit unique properties in diverse reactions [145]. Nevertheless, achieving precise control over the geometric shape of nanoparticles in the exsolution process is not commonly observed. Unlike the wet chemical colloidal method, the exsolution process occurring through gas reactions involves no use of capping agents that selectively attach to the surface and stabilize specific crystal facets. A notable study reported that CO gas plays a pivotal role in the formation of (100)-faceted Ni particles; however, the mechanism of this phenomenon remains unclear [59]. At the present stage, there is a pressing need for both a comprehensive understanding of the shape-shifting phenomenon and the development of strategies to effectively control the crystal facets of exsolved particles.

### Novel Strategies

In recent years, numerous cutting-edge studies have introduced innovative approaches to enhance exsolution behaviors through various methods, including physical, chemical, and electrochemical techniques. Enhanced exsolution plays a critical role in achieving high performance across diverse applications. However, the detailed mechanism underlying these advanced strategies remains unclear. Specifically, it is crucial to elucidate the kinetics involved in the significantly shortened growth time observed during electrochemical switching or thermal shock. Further investigations are required to deepen our mechanistic understanding of the factors contributing to enhanced exsolution behaviors.
